# Comparative transcriptomic analysis of contrasting hybrid cultivars reveal key drought-responsive genes and metabolic pathways regulating drought stress tolerance in maize at various stages

**DOI:** 10.1371/journal.pone.0240468

**Published:** 2020-10-15

**Authors:** Songtao Liu, Tinashe Zenda, Jiao Li, Yafei Wang, Xinyue Liu, Huijun Duan

**Affiliations:** 1 State Key Laboratory for Crop Improvement and Regulation, Hebei Agricultural University, Baoding, China; 2 North China Key Laboratory for Crop Germplasm Resources of the Education Ministry, Hebei Agricultural University, Baoding, China; 3 Department of Crop Genetics and Breeding, College of Agronomy, Hebei Agricultural University, Baoding, China; ICAR-Indian Institute of Agricultural Biotechnology, INDIA

## Abstract

Drought stress is the primary environmental factor that negatively influences plant growth and yield in cereal grain crops such as maize (*Zea mays* L.). Crop breeding efforts for enhanced drought resistance require improved knowledge of plant drought stress responses. In this study, we applied a 12-day water-deficit stress treatment to maize plants of two contrasting (drought tolerant ND476 and drought sensitive ZX978) hybrid cultivars at four (V12, VT, R1, and R4) crop growth stages and we report key cultivar-specific and growth-stage-specific molecular mechanisms regulating drought stress responses in maize. Based on the transcriptome analysis, a total of 3451 and 4088 differentially expressed genes (DEGs) were identified in ND476 and ZX978 from the four experimental comparisons, respectively. These gene expression changes effected corresponding metabolic pathway responses related to drought tolerance in maize. In ND476, the DEGs associated with the ribosome, starch and sucrose metabolism, phenylpropanoid biosynthesis and phenylpropanoid metabolism pathways were predominant at the V12, VT, R2, and R4 stages, respectively, whereas those in ZX978 were related to ribosome, pentose and glucuronate interconversions (PGI), MAPK signaling and sulfur metabolism pathways, respectively. MapMan analysis revealed that DEGs related to secondary metabolism, lipid metabolism, and amino acid metabolism were universal across the four growth stages in ND476. Meanwhile, the DEGs involved in cell wall, photosynthesis and amino acid metabolism were universal across the four growth stages in ZX978. However, K-means analysis clustered those DEGs into clear and distinct expression profiles in ND476 and ZX978 at each stage. Several functional and regulatory genes were identified in the special clusters related to drought defense response. Our results affirmed that maize drought stress adaptation is a cultivar-specific response as well as a stage-specific response process. Additionally, our findings enrich the maize genetic resources and enhance our further understanding of the molecular mechanisms regulating drought stress tolerance in maize. Further, the DEGs screened in this study may provide a foundational basis for our future targeted cloning studies.

## Introduction

Field grown crops inevitably endure a plethora of abiotic stresses during their growth and development process, including drought, heat, cold, salt and metal toxicity [1]. Among these abiotic factors, drought is the primary threat to global crop production and food security. As a result of global warming and climate change, drought has increased in its frequency, severity and geographical spread [2]. More startling, climate models predict more frequent and severe extreme weather events, including drought, in many regions for the next decades, which will definitely hamper crop productivity [3]. This would consequently lead to an imbalance between rising human population and increased (and changing) dietary requirements, thereby posing a serious threat to global food and nutrition security. Therefore, much research is required to systematically reveal drought response mechanisms in crops, which will improve crop performance under water-stressed conditions and assist to achieve agricultural sustainability and world food security.

Maize (*Zea mays* L.) is one of the major cereal grain crops providing a stable food supply worldwide. According to the data published in the Food and Agriculture Organization of the United Nations (FAO), in 2017, the maize harvest area was 1.97×10^8^ ha, and production was 1.13 ×10^10^ ton around the world. At national level, there were 4.24×10^8^ ha of harvest area and 2.59 ×10^9^ ton of production, ranking first ahead of rice (*Oryza sativa* L.) and wheat (*Triticum aestivum* L.) in China [4]. Unfortunately, the northern region of China, including Hebei Province, is the country`s largest maize producing area, located in arid and semiarid regions and is constantly faced with great drought disaster risks [5]. Yet, maize crop is susceptible to water-stressed conditions during its whole life, with the vegetative and reproductive stages requiring comparably more water than the seedling stage [6]. In short to medium-term, supplementing water through irrigation may cushion the crops against the drought effects. However, more sustainably, understanding the molecular mechanisms governing maize drought stress responses at various growth phases is critical for the crop improvement aimed at enhancing maize drought resistance and reduction of production risks.

Being sessile in nature, crops harbour complex regulatory mechanisms to respond to water deficit, such as enhanced signal perception, transcription regulation, gene expression modulation and metabolism reprogramming [7]. Within this complex machinery, stress perception is arguably the first step to recognize the water-deficit cues, followed by signal transduction and gene expression regulation, that at last induce the intricate metabolic and physiological changes to battle water deficit stress [8,9]. Abscisic acid (ABA) is the best-known hormone messenger related to drought signaling, which not only play vital roles in signal transduction, but also serves as a regulator in transcriptional regulatory networks [10]. Transcription factors (TFs) are also considered key players in plant growth, development and abiotic stress responses [11]. The highly drought-responsive TFs such as myeloblastosis (MYB); NAM, ATAF, CUC (NAC); and ABA-responsive element binding factors (ABFs) follow an ABA-dependent pathway. Moreover, drought-responsive element binding (DREB) factors have gained much attention in drought response via ABA-independent pathway [12]. Other gene groups, such as protein kinases, antioxidant enzymes, late embryogenic abundant (LEA) and heat shock proteins (HSPs) also play critical roles in response to water deficit stress [13,14]. Lang and Buu [15] have found that adaptation to drought is polygenic with intricate changes in metabolism. Multiple metabolic processes such as CO_2_ assimilation, carbohydrates metabolism and amino acid biosynthesis are disrupted by drought stress, which ultimately reduces the biomass accumulation rate [16]. In recent years, a great number of drought stress related metabolic pathways have been identified and thus, analysis of plant metabolic pathway enrichment of genes under drought stress has become an important strategy in screening out key drought stress response mechanisms in various crops [17].

In recent years, the development of '-omics' and high-throughput sequencing technologies such as Solexa/ Illumina and digital gene expression analysis has provided for efficient and powerful approaches to investigate genome-wide gene expression changes in response to abiotic stresses [18]. Transcriptome analysis using RNA-sequencing (RNA-seq) has offered us convenience in detecting novel genes in a tissue- or cell-specific manner and enabled us to identify transcriptomic changes under certain biological conditions [19]. RNA-seq has been successfully applied in global gene expression profiling in maize response to drought stress [20–22]. Despite all this progress, however, most of these transcriptome researches in maize have focused on a single stage separately, especially the seedling [22], vegetative [20] or reproductive [19] stages. Very few maize drought stress response studies [23–25] have been performed focusing on different crop growth stages within the same study. Therefore, investigating maize plants responses to drought stress at different growth phases within a single study could give a better picture on the whole-plant, cellular and growth-stage-specific responses to drought stress.

Traditionally, the differentially expressed genes (DEGs) were identified based on the gene expression levels between experimental groups. Then, according to a level of significance, we acquire a substantial number of candidate genes. This classical method provides an easy way to conduct gene expression analysis. However, the complexity of biological systems is also related to the intricate dynamics that they exhibit. Thus, there is a great need to evolve analytical methodology to discover distinct expression patterns in gene expression data. To this end, cluster analysis is the common computational approach for analyzing gene expression data and is largely recognized as a useful exploratory tool [26]. According to the hypothesis that genes belonging to a particular metabolic pathway should be co-regulated and show the resemblant expression level, clustering techniques classify DEGs with the similar expression patterns into groups (clusters) that may be associated in terms of their complex biological functions. Such cluster-based analysis has been used in studying plants response to abiotic stresses [27].

In order to clarify the fundamental cultivar-specific and developmental-stage specific molecular mechanisms underpinning maize drought stress response, in the present study, we used (in a comparative analysis) two maize hybrid cultivars with contrasting drought tolerance [tolerant line Nongdan 476 (ND476) and sensitive line Zhongxin 978 (ZX978)] grown in the field under well-watered and water-deprived conditions. A total of 48 leaf tissue samples collected from the two hybrid cultivars (ND476 and ZX978), two conditions (control and drought treatment) and four crop growth stages, from the vegetative to reproductive, (viz., V12, VT, R2 and R4) were used for c-DNA library construction employed in the subsequent Illumina RNA-seqand comparative analyses. Firstly, to identify drought responsive genes and genotypic differences, differential expression analysis between treatments and cultivars was conducted. Secondly, we visualized the key DEGs involved in metabolic pathways and revealed the transcription factor (TF) changes in response to drought stress. Finally, clustering analysis was performed from the drought-induced transcriptome profiles of two hybrid cultivars across four growth stages to classify co-regulated genes within the biological processes category after drought treatment. Overall, our results could provide a global view of the regulatory molecular mechanisms of drought-stress response. Additionally, our findings could aid in mining drought responsive genes and molecular breeding and genetic engineering of new drought-tolerant maize cultivars.

## Results

### Analysis of RNA-sequencing and transcriptome profiling results

In order to explore genes responsive to drought stress in the leaf tissues of two maize genotypes-ND476 (drought-tolerant) and ZX978 (drought-sensitive), global gene expression was surveyed by next-generation sequencing. The transcriptome of each hybrid line was analyzed at V12, VT, R2 and R4 stages, both for the well-watered and drought-stressed conditions. Resultantly, we obtained 48 samples in total, which included three biological replicates for each time point and condition. The raw sequencing data were deposited into the NCBI Sequence Read Archive (SRA, Accession SPR212360). After filtering, Illumina RNA-seq yielded 240.09 million clean reads of 150bp length, with about 50.02 million reads on average from each sample. Of these reads, 72.72–89.33% could be mapped onto unique positions on the maize reference genome (ZmB73_Ref-Gen_v4). The Q30 score and GC percentages of all the libraries were above 94.33% and 53.41%, respectively, which met the requirements for further analysis (S1 Table).

To evaluate the relatedness of the 48 transcriptome samples, cluster analysis for ND476 and ZX978 samples was performed using the fragments per-kilobase of the exon model per million mapped reads (FPKM) method. The cluster results showed highly correlation between samples and clear separation of each growth stage (S2 Table). Furthermore, principal component analysis (PCA) was conducted to assess the similarities and differences between samples. PCA analysis results of ND476 and ZX978 presented the same tendency as the cluster results (S1 Fig). The above results confirmed that our sequencing data was reproducible and reliable, which could be used for further analysis.

### Analysis of gene expression changes between two contrasting maize genotypes across different drought treatments and different crop growth stages

**Fig 1:**
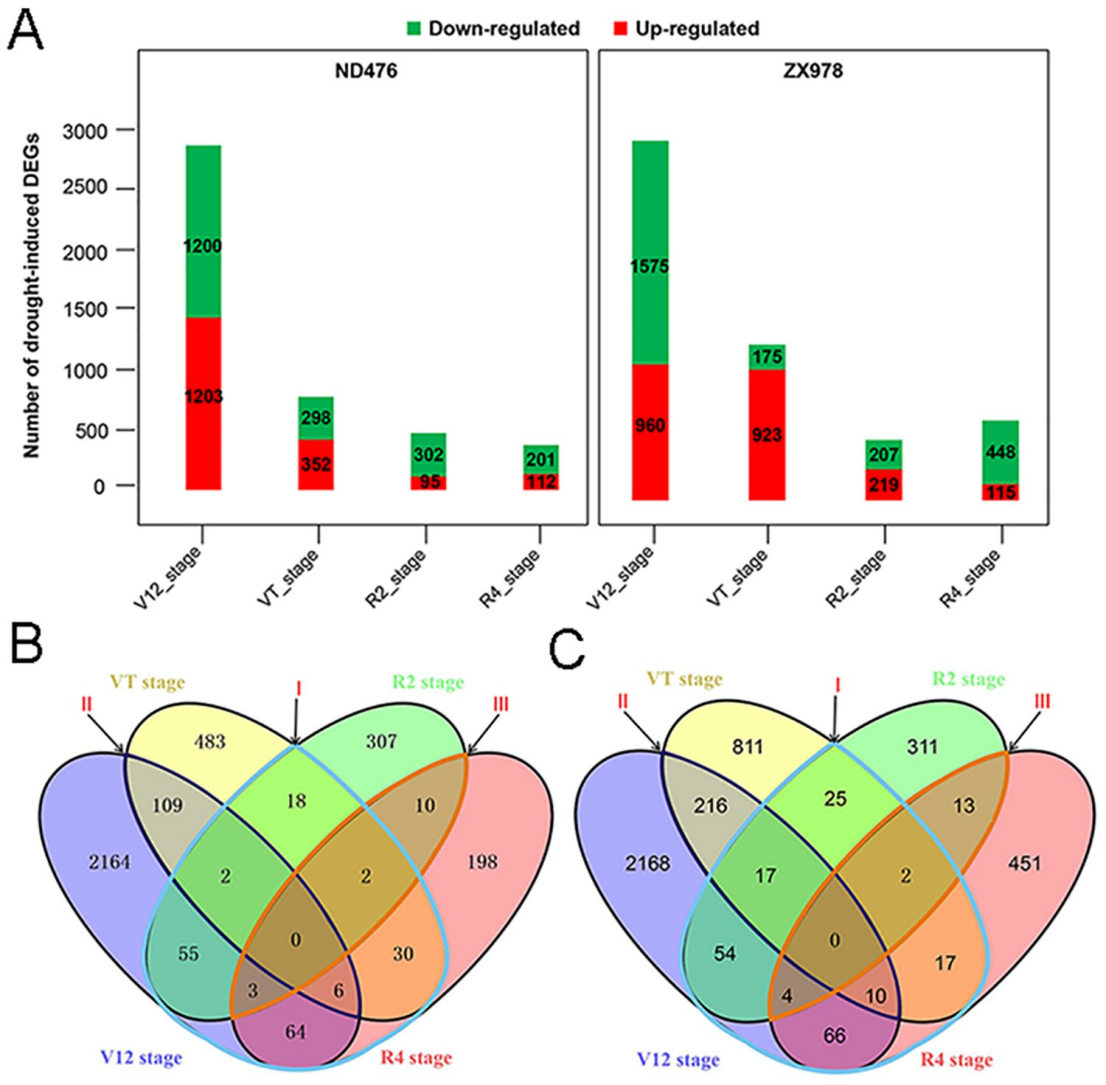
Differentially expressed genes (DEGs) analysis in two contrasting maize hybrid lines ND476 and ZX978. (A) Number of DEGs identified in each experimental stage (B) Venn diagram analysis of ND476 DEGs across four crop growth stages (C) Venn diagram analysis of ZX978 DEGs across four growth stages.

To identify genes differentially expressed between well-watered and water-deficit conditions, four pairwise comparisons of control versus drought of each hybrid line at V12, VT, R2 and R4 stages were generated. Differences in DEGs abundance across different treatment points, from V12 to R2 stages, in both the tolerant and sensitive genotypes were evident, which indicated s a spatio-temporal dynamic pattern of maize response to water-deficit conditions. The number of DEGs was highest at the V12 stage, both in ND476 (2403 DEGs; 1203 up-regulated and 1200 down-regulated) and in ZX978 (2535 DEGs; 960 up- and 1575 down-regulated) after drought treatment. At the VT stage, 650 DEGs (352 up- and 298 down—regulated) were identified in ND476, whist 1098 DEGs (923 up- and 175 down-regulated) were identified in ZX978 in response to drought stress. As for the R2 stage, 397 DEGs (112 up-and 201 down-regulated) were observed in ND476, whereas 426 DEGs (219 up- and 207 down-regulated) were identified in ZX978 under drought stress conditions. The R4 stage of ND476 had the smallest number of DEGs (313 DEGs; 112 up-and 201 down-regulated) compared with 563 DEGs observed in ZX978 (115 up- and 448 down-regulated) (Fig 1A). The DEGs number of ZX978 was higher than that in ND476 at each treatment stage. This may suggest that probably because of its conserved better water-deficit endurance capacity, the tolerant line ND476 perceived the drought treatment conditions as moderate and instituted a limited transcriptome response whereas the susceptible line ZX978, because of its lack of inherent drought endurance capacity, may have perceived the same drought conditions as severe and hence activated correspondingly boundless transcriptome response. In total, we identified 3451 and 4088 DEGs in ND476 and ZX978 cultivars, respectively, in response to drought (Fig 1B and 1C). Among these identified DEGs, 2164, 483, 307, and 198 were growth stage-specific to V12, VT, R2 and R4 stages, respectively, in ND476 (Fig 1B). Further analysis of the tolerant line ND476 DEGs showed that 180 genes responding to drought at the vegetative (V12 and VT) stages were also differentially expressed at the reproductive (R2 and R4) stages (Fig 1B label I). A set of 117 DEGs were shared between V12 and VT stages, whereas 15 genes were shared between R2 and R4 stages of ND476 after drought treatment (Fig 1B label II, III). Similarly, a great number of DEGs were growth stage-specific in the sensitive genotype ZX978. After drought treatment, 2168, 811, 311 and 451 responsive genes were uniquely expressed at the V12, VT, R2 and R4 stages, respectively (Fig 1C). There were 195 DEGs shared between the vegetative (V12 and VT) and reproductive (R2 and R4) stages of ZX978 after drought treatment (Fig 1C label I). In ZX978, 243 DEGs were shared between the V12 and VT stages, whist 19 DEGs were shared between the R2 and R4 stages after drought treatment (Fig 1C label II, III).

### DEGs annotation and functional categorization

In order to assign putative biological functions to the DEGs, gene ontology (GO) annotation was performed. We considered the DEGs related to biological process (BP) category to be the most informative in the context of drought stress response, and we paid much attention to the DEGs expressed in this category. Our GO analysis results showed that a great number of DEGs in the BP categories were shared among the four crop growth stages in both ND476 and ZX978 hybrid cultivars. In both hybrid cultivars, the most represented BP subcategories were metabolic process (GO: 0008152), cellular process (GO: 0009987) and single-organism process (GO: 0044699). Additionally, DEGs associated with response to stimulus (GO: 0050896) were prominent in drought stress response (S2 Fig). Our comparative analysis revealed that the numbers of DEGs in sensitive line ZX978 were greater than those in the tolerant line ND476 at each crop growth stage. However, the percentages of DEGs annotated to GO terms were less in ZX978 than those in ND476 (S2 Fig). Here, our results may suggest that the significant difference in drought tolerance between the two hybrid cultivars could be emanating from the difference in the number of genes enriched in each of those shared GO terms.

### Analysis of identified drought stress regulatory transcription factors

Regulatory TFs play a vital role in eliciting response to drought stress. In our study, several transcription factor families were detected as differentially expressed, including bHLH, bZIP, HSF, NAC, MYB-related, MYB and WRKY. Some of these DEGs were more expressed in the drought-tolerant and others in the drought-sensitive genotypes (Table 1). Amongst these TFs, the number of bHLH was the highest and identified in both hybrid cultivars across the four crop growth stages. Additionally, HB, AP2/EREBP, C_2_H_2_, MYB and WRKY were observed to be differentially expressed in both hybrid cultivars at every crop growth stage. However, several TFs including HAP2, HAP3, HDA and DR1 were identified uniquely in tolerant line ND476, whereas HMG and AtSR TFs were specifically expressed in the sensitive line ZX978. Taken together, the modulation of different TFs belonging to various families suggests the complexity of regulatory networks related to maize drought stress response, even though further in-depth research would be needed to elucidate these TFs`exact functions in drought response.

**Table 1:** TF gene families identified in two drought-tolerance contrasting maize genotypes ND476 and ZX978.

	Gene number in tolerant ND476				Gene number in sensitive ZX978			
	Crop growth stage				Crop growth stage			
TF family	V12 stage	VT stage	R2 stage	R4 stage	V12 stage	VT stage	R2 stage	R4 stage
AP2/EREBP	7	4	2	1	7	5	1	6
ARF	7	3	0	1	1	0	0	0
ARR	2	1	1	1	3	1	0	1
AS2	1	2	3	0	4	2	0	1
AtSR	0	0	0	0	1	0	0	1
Aux/IAA	4	3	0	0	6	1	0	1
bHLH	12	6	3	2	13	3	3	3
bZIP	3	3	3	2	7	4	0	1
C2C2(Zn) CO-like	3	0	0	0	3	1	0	0
C2C2(Zn) DOF	2	1	0	0	5	1	1	0
C2C2(Zn) GATA	2	1	0	1	1	0	0	2
C2H2	9	1	2	1	3	1	1	7
C3H	2	1	0	2	2	0	0	0
DR1	1	0	0	0	0	0	0	0
GARP	2	4	1	2	5	0	1	0
GRAS	1	0	1	0	0	1	0	0
HAP2	2	0	1	0	0	0	0	0
HAP3	2	0	1	0	0	0	0	0
HAP5	0	0	0	1	1	0	0	0
HB	15	2	1	4	9	6	3	1
HDA	1	0	0	0	0	0	0	0
HMG	0	0	0	0	1	0	0	0
HSF	1	3	0	0	2	2	3	0
JUMONJI family	2	2	1	0	0	0	0	1
MADS box	5	0	1	1	2	2	1	1
MYB	7	6	2	2	16	3	2	1
MYB-related	4	1	1	1	4	0	0	1
NAC	6	5	0	1	6	0	3	1
PHD	1	1	0	0	2	1	0	0
putative transcription regulator	15	0	2	0	2	0	2	2
SET-domain	4	1	0	0	3	2	2	0
TCP	2	1	0	0	3	0	0	0
Trihelix	4	2	0	0	0	0	1	0
WRKY	6	2	1	1	4	3	5	6

### KEGG pathway enrichment analysis of the DEGs

**Fig 2:**
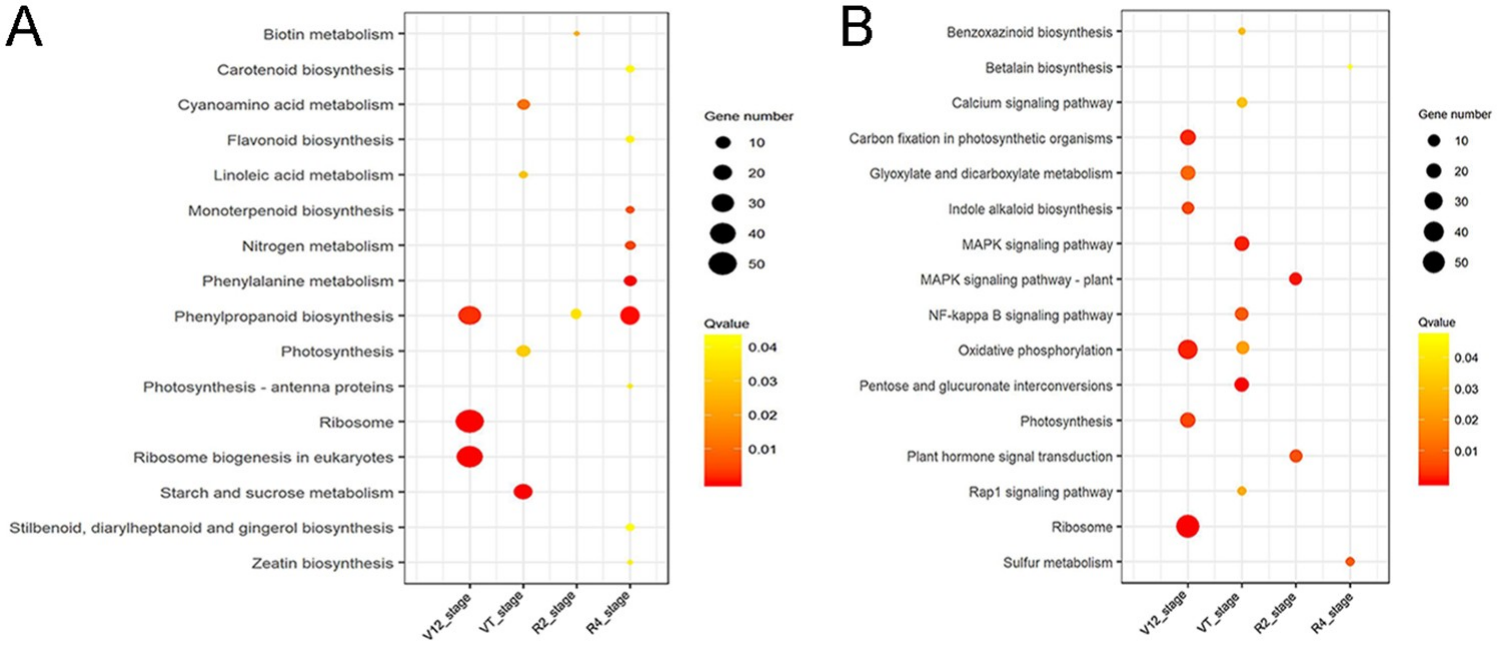
KEGG pathway enrichment analysis of DEGs. Sub-figures show the most significantly enriched KEGG pathways in (A) ND476; and (B) ZX978 genotypes across the four crop growth stages.

For both maize genotypes, the DEGs of each stage were also subjected to KEGG (Kyoto Encyclopedia of Genes and Genomes) pathway enrichment analysis (Q-value < 0.05) to investigate the functional fate of those identified drought responsive DEGs in various metabolic pathways. The DEGs were assigned to 16 KEGG pathways amongst the four crop growth stages in both maize lines. Among these, there were 12 and 9 metabolism processes related pathways in ND476 and ZX978, respectively (Fig 2). The main metabolism related pathways identified in the two hybrid cultivars were 'ribosome' and 'photosynthesis'. In the tolerant line ND476, 'phenylpropanoid biosynthesis' was significantly enriched at the V12, R2 and R4 stages. As for the VT stage, 'starch and sucrose metabolism', 'cyano amino acid metabolism' and 'linoleic acid metabolism' were the top most enriched pathways. Notably, the DEGs involved in secondary metabolites biosynthesis and nitrogen metabolism pathways (phenylalanine, flavonoid, nitrogen metabolism, etc.) were specifically enriched in ND476 at the R4 stage. Contrastingly, in the sensitive line ZX978, energy metabolism related (oxidative phosphorylation, carbon fixation in photosynthetic organisms, etc.) pathways were highly enriched at the V12 stage, whist 'sulfur metabolism' and 'betalain biosynthesis' were dominant at the R4 stage. Additionally, the pathways involved in signal transduction such as 'MAPK signaling', 'NF-kappa B signaling', 'Rap1 signaling' and 'calcium signaling' pathways were highly enriched in ZX978 at the VT stage, whereas 'plant hormone signal transduction' and 'MAPK signaling pathway—plant' were apparent at the R2 stage. These observations indicated that the DEGs enriched in metabolism (especially nitrogen, secondary metabolites biosynthesis, energy and signal transduction) related pathways are critical for the identification of drought stress response related genes.

### Analysis of the dynamic gene expression patterns of the two maize hybrid cultivars

**Fig 3:**
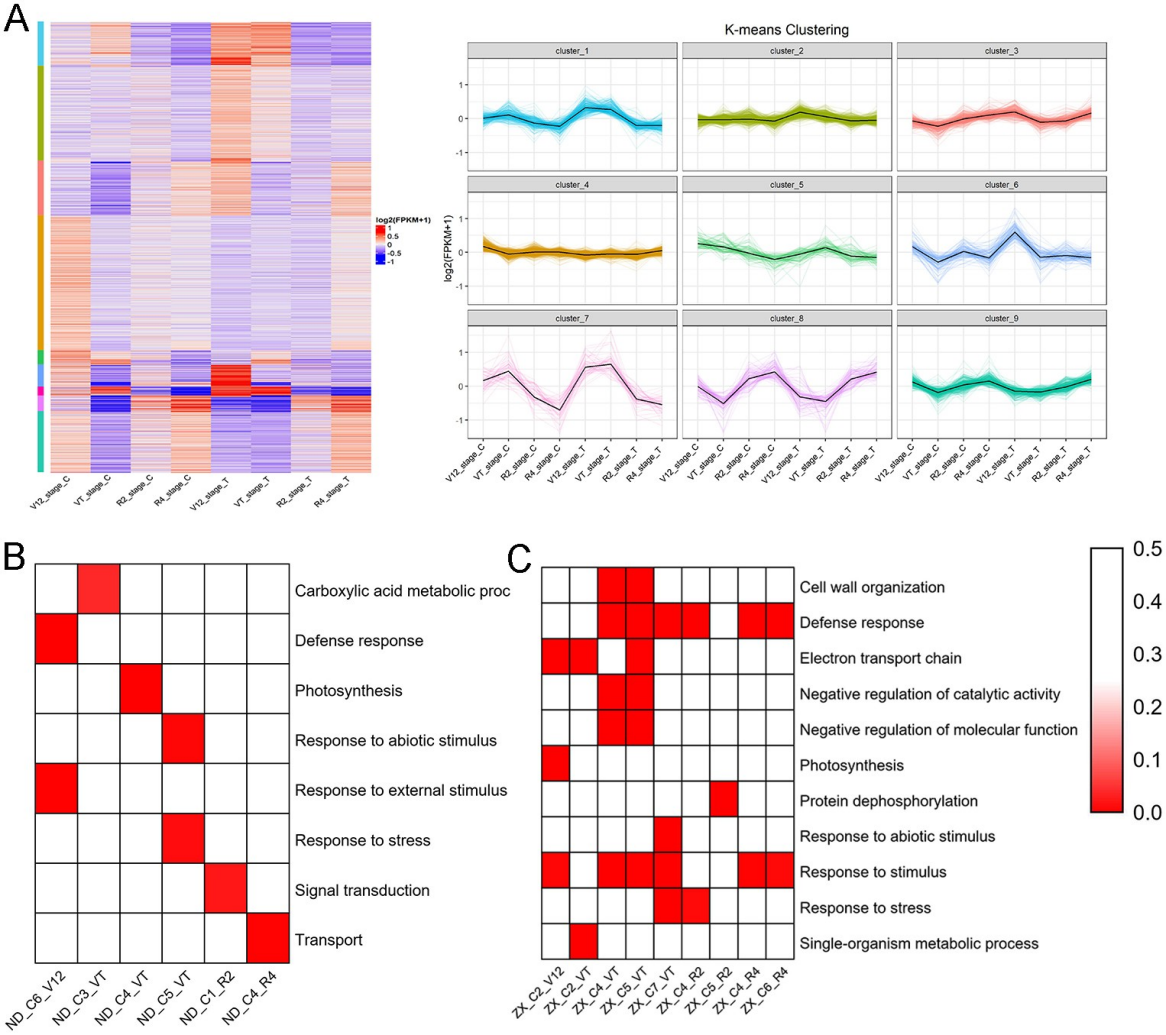
Cluster analysis of DEGs during drought treatment. (A) Heat map illustrating the expression profiles of the DEGs of ND476 at the four growth stages. The bars on the left side represent the hierarchical clustering analysis results while the nine clusters on the right side show the analysis results of the gene expression profiles with the K-means algorithm. (B) GO enrichment analysis of special clusters identified in drought-tolerant cultivar ND476. (C) GO enrichment analysis of special clusters identified in drought-sensitive cultivar ZX978.

To investigate the expression patterns of DEGs identified in two hybrid cultivars across four crop growth stages under well-watered and drought conditions, K-means clustering analysis was conducted. This enabled us to identify gene clusters with distinct expression profiles in ND476 and ZX978 cultivars at each stage. In the drought-tolerant line ND476, a total of 2403 DEGs of the V12 stage were divided into nine clusters, among which cluster 6, enriched for defense-response-related functions, showed significantly high expression after drought treatment (Fig 3A, S3 Table). At the VT stage, cluster 3 included 84 DEGs linked to carboxylic acid metabolic process which exhibited slightly increased expression after drought treatment. Meanwhile, we observed that 57 stage-specific and photosynthesis related DEGs in cluster 4 displayed high expression, but 48 stage-specific and response-to-stress related DEGs in cluster 5 showed low expression under well-watered and drought treatment conditions (Fig 3B, S3A Fig, S3 Table). After drought treatment, the DEGs annotated to R2 stage exhibited largely decreased expression, particularly 140 genes of cluster 1 that are involved in signal transduction (Fig 3B, S3B Fig, S3 Table). As for the R4 stage, cluster 4 and transport related DEGs comprised 76 stage-specific genes showing high expression under well-watered and drought treatment conditions (Fig 3B, S3C Fig, S3 Table).

Specific expression patterns and biological processes enrichment were also identified in sensitive hybrid line ZX978. Cluster 2 DEGs (comprising 112 DEGs related to 'electron transport chain', 'photosynthesis' and 'response to stimulus' functions) displayed decreased expression after drought treatment at the V12 stage (Fig 3C, S4A Fig, S4 Table). As for the VT stage, 121 DEGs in cluster 2 showed slightly increased expression tendency and got enriched in 'electron transport chain' and 'single-organism metabolic process' related functions. We also observed high expression of 166 stage-specific DEGs in cluster 5 and 99 stage-specific DEGs in cluster 6 at the VT stage after drought treatment. These two clusters harbored DEGs related to 'negative regulation of molecular function', 'cell wall organization', 'negative regulation of catalytic activity', 'defense response' and 'response to stimulus' functions. Moreover, cluster 7 DEGs (involved in 'response to abiotic stimulus', 'response to stress', 'response to stimulus' and 'defense response') showed decreased abundance after drought treatment as compared to under well-watered conditions at the V12, R2 and R4 stages, but exhibited increased abundance at the VT stage (Fig 3C, S4B Fig, S4 Table). Cluster 4 comprised 37 DEGs linked to defense response and they exhibited low expression after drought treatment. However, cluster 5 comprised 56 DEGs related to protein dephosphorylation and they displayed high expression after drought treatment at the R2 crop growth stage (Fig 3C, S4C Fig, S4 Table). Generally, cluster 4 and cluster 6 DEGs, enriched for defense-response and response-to-stress functions, showed significantly high expression under well-watered conditions, but low expression after water-limited conditions (Fig 3C, S4D Fig, S4 Table).

### Overview of the DEGs involved in metabolism processes

**Fig 4:**
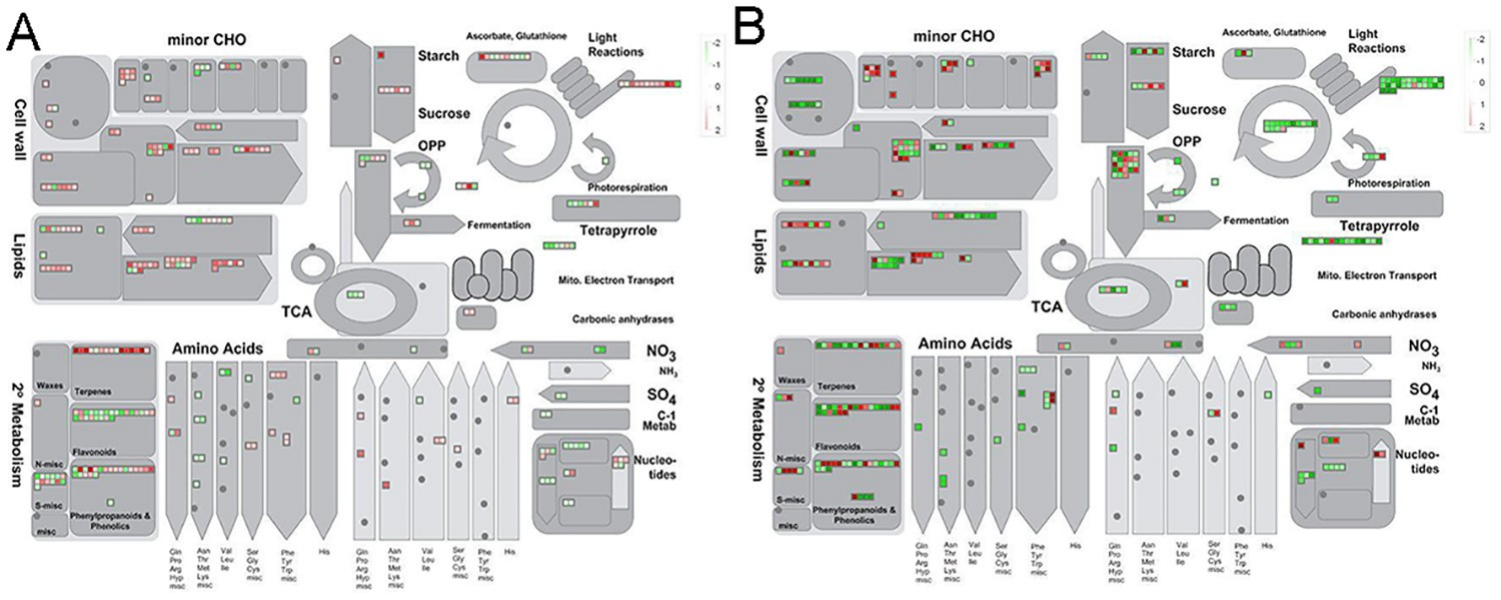
Mapman overview of maize genotypes`metabolic responses to drought stress. (A) The DEGs of ND476, and (B) DEGs of ZX978 identified at the V12 growth stage after drought treatment.

**Fig 5:**
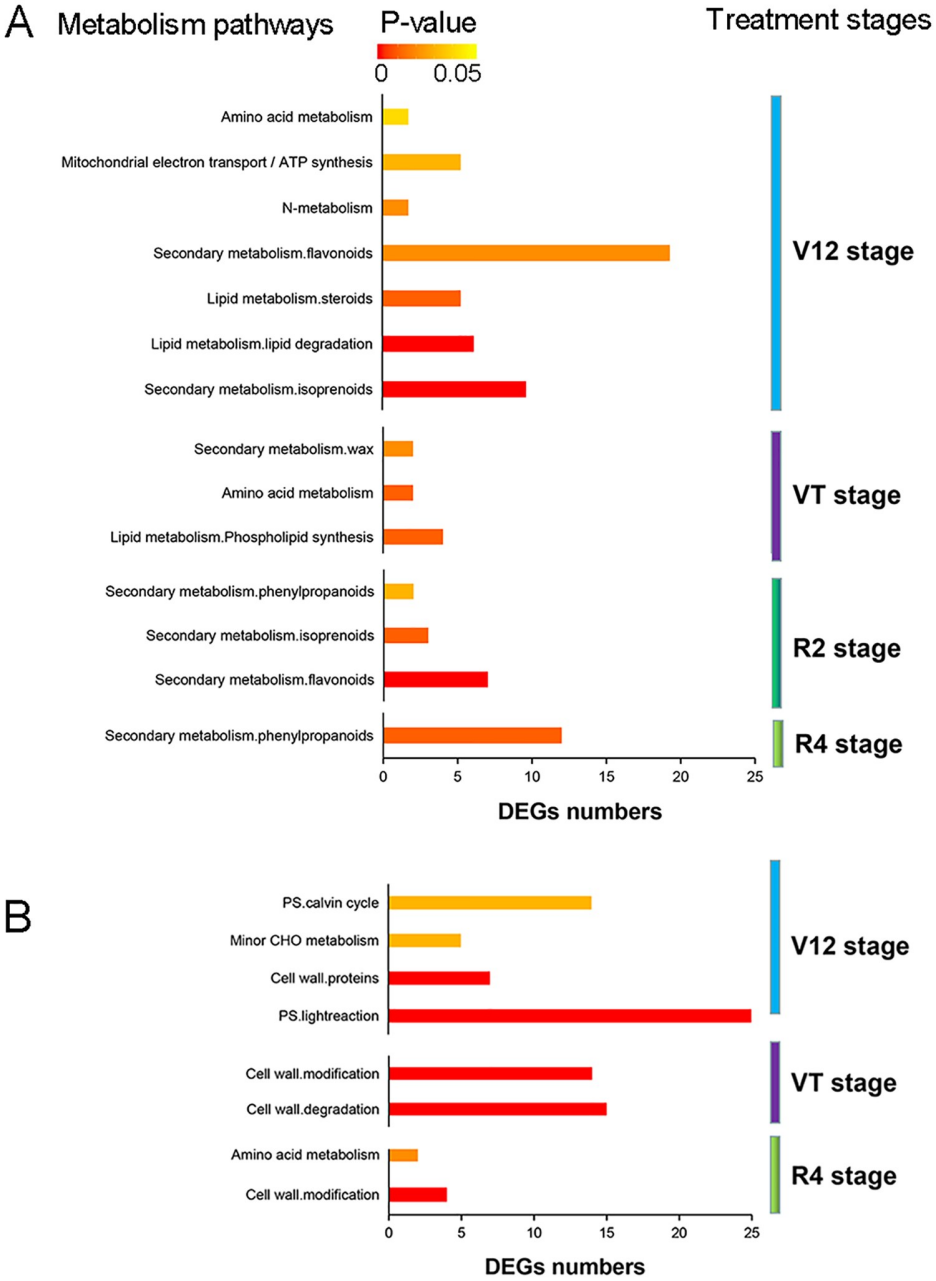
Metabolic pathways enrichment analysis of DEGs. Most significantly enriched metabolic pathways in (A) drought tolerant hybrid cultivar ND476, and (B) drought sensitive hybrid cultivarZX978, based on the hypergeometric test, *p* < 0.05.

The GO and KEGG enrichment analysis of DEGs implied that gene expression responses to drought stress lead to altered metabolism. Therefore, an overview of the metabolic processes regulated by drought was performed by MapMan (Fig 4A and 4B, S5 Fig). The complete DEGs annotated to metabolism processes were summarized in S5 and S6 Tables for ND476 and ZX978, respectively. In the drought tolerant hybrid line ND476, DEGs involved in secondary metabolites (isoprenoids, flavonoids, etc.) biosynthesis, lipid metabolism (lipid degradation, steroids, etc.), N-metabolism, amino acid metabolism, and mitochondrial electron transport / ATP synthesis were highly enriched in response to water-deficit treatment at the V12 stage (Figs 4A and 5A). At the VT stage, lipid metabolism (phospholipid synthesis), amino acid metabolism and secondary metabolism of wax were mainly enriched (Fig 5A, S5A Fig). Moreover, secondary metabolites biosynthesis processes such as isoprenoids, flavonoids and phenylpropanoids biosynthesis were highly enriched at the R2 and R4 stages (Fig 5A, S5C and S5E Fig).

On the other hand, in the sensitive hybrid line ZX978, there were increased numbers of responsive genes in all pathway categories, with several DEGs enriched in cell wall proteins and photosynthesis (light reaction, Calvin cycle, etc.) processes at the V12 stage (Figs 4B and 5B). Meanwhile, the DEGs involved in cell wall degradation and modification were highly enriched at the VT stage (Fig 4B, S5B Fig). The DEGs identified at the R2 stage were not significantly enriched in any metabolism related pathways (S5D Fig), whereas the DEGs related to cell wall modification and amino acid metabolism were highly enriched at the R4 stage (Fig 4B, S5F Fig).

### Quantitative real-time PCR (qRT-PCR) validation

**Fig 6:**
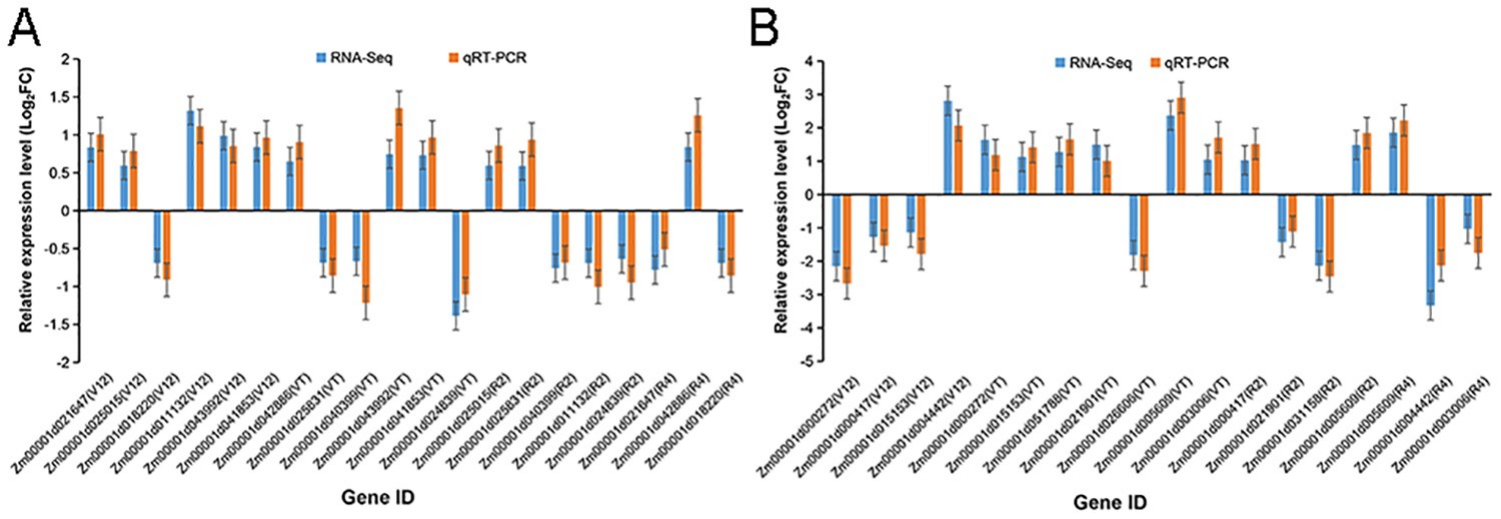
qRT-PCR validation of the RNA-seq data for the 20 randomly selected maize leaf DEGs. (A) DEGs identified in hybrid cultivar ND476, and (B) DEGs identified in hybrid cultivar ZX978. Error bars represent the SE (n = 3).

To further verify the expression profiles of the genes in our RNA-seq analyses, a total of 20 DEGs were randomly selected for qRT-PCR using leaf samples originally used for Illumina RNA-seq. We also converted the gene expression into log 2 (fold change), which we then used to analyze the DEGs. Resultantly, the expression levels of these qRT-PCR selected genes displayed high similarity to the gene expression tendencies of the RNA-seq data (Fig 6, S7 Table). A correlation coefficient (R^2^) of the log 2 (fold change) between qRT-PCR and RNA-seq implied a significantly high similarity (R^2^ > 0.9) between the transcripts and gene expression levels after drought treatment (S6 Fig), endorsing the reliability of the RNA-seq data.

### Physiological responses of ND476 and ZX978 maize hybrid cultivars to drought stress at different crop growth stages

**Fig 7:**
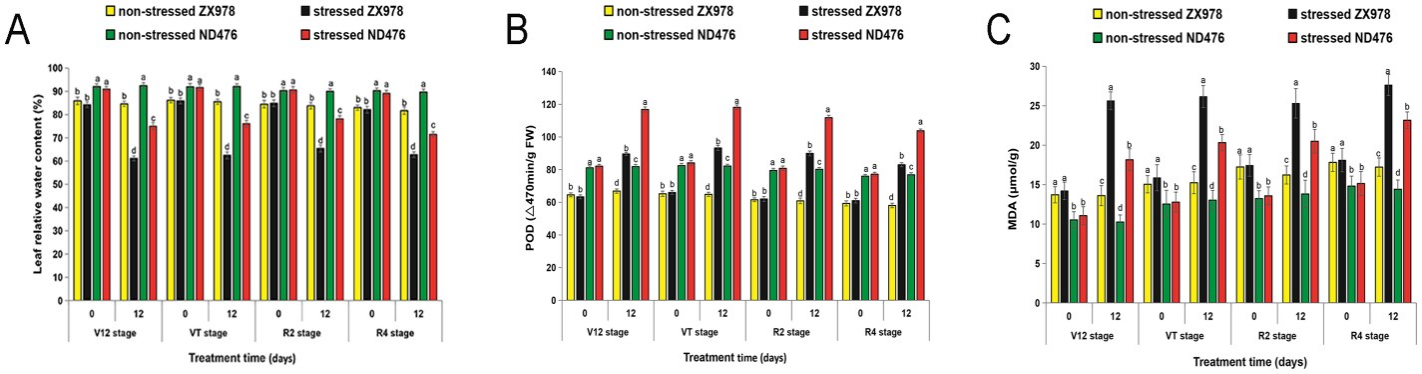
Physiological responses of ND476 and ZX978 maize hybrid cultivars to a 12-day drought treatment exposure at various growth stages. (A) Leaf RWC (B) POD activity and (C) MDA content. Data are presented as means ±SE (n = 3). Different letters above bar graphs show significant difference (p < 0.05) among treatments at a given treatment time point.

To augment the molecular analysis, we evaluated the two maize genotypes`physiological responses to drought stress, by determining leaf relative water content (RWC), guaiacol peroxidase (POD) enzyme activity and malondialdehyde (MDA) content at four different crop growth stages. As expected, both maize hybrid lines did not show significant differences in physiological parameters under control conditions. However, drought stress led to changes in physiological parameters at different stages (Fig 7). Drought treatment caused significant (p < 0.05) decline in RWC with increasing treatment exposure duration in both cultivars and at all the four (V12, VT, R2 and R4) stages. However, this rate of decline was comparably sharp in ZX978 than in ND476 at most stages (Fig 7A). Therefore, ND476 could be considered as ‘drought tolerant’ and ZX978 ‘drought sensitive’ to water deficit. Drought stress significantly increased the POD activity in both maize lines. Notably, ND476 maintained comparably higher POD activity than ZX978 under water deficit stress at any given time point (Fig 7B). MDA content was increased in both maize lines under drought stress, but it was much greater in the sensitive line ZX978 than in the tolerant line ND476. Additionally, our results showed that MDA content got amplified with the increasing number of stress exposure days (Fig 7C). Overall, our findings indicated that under field drought stress conditions and at different growth stages, RWC and POD enzyme activity were significantly higher in the tolerant genotype ND476 than in sensitive genotype ZX978, whereas MDA content was greater in ZX978 than in ND476.

## Discussion

Drought stress tolerance in plants is a complex process, with various responses being instituted at the physiological, biochemical, cellular and molecular levels [7]. To obtain comprehensive understanding of the molecular mechanisms underpinning drought tolerance in maize, here, we used RNA-seq approach to perform a comparative analysis of two hybrid cultivars contrasting in drought tolerance across four crop growth stages. Our investigation identified cultivar-specific and growth stage-specific genes regulating drought stress response in maize. Our findings offer new paradigms of drought stress regulatory mechanisms in maize at the transcriptome level, as well as providing a foundational basis for molecular designing of new drought tolerant cultivars.

### Drought stress caused differential physiological changes in two contrasting maize genotypes

The adaptive response of plants to drought stress involves a series of physiological and biochemical changes [28]. Here, our physiological analysis results showed that the two maize cultivars performed differently under drought stressed conditions. Leaf RWC decreased significantly greater in ZX978 than in ND476 under drought stress conditions, and at almost all stress exposure time periods (Fig 7A). This could have helped ND476 genotype to perform physio-biochemical processes comparably more efficient that ZX978 under drought stress conditions. This is in conformity with a similar study by Moussa and Abdel-Aziz [29]. In plants responding to abiotic stresses, POD is crucial in scavenging ROS and protecting the cellular membrane from peroxidation. Usually, the peroxidases serve as detoxifying enzymes and remove toxic reductants [30]. Here, our results showed that the abundance of POD in both maize cultivars increased in response to drought treatment, with ND476 genotype exhibiting higher POD levels as compared to ZX978 (Fig 7B).

It is widely acknowledged that cell membrane integrity and stability maintenance under water deficit stress is a major component of drought tolerance [29]. The rise in MDA content under stress conditions suggests that drought stress could induce membrane lipid peroxidation by means of ROS. Therefore, cell membrane stability, reciprocal to cell membrane injury, is a physiological index widely used for the evaluation of drought tolerance [31]. In the present study, the MDA content was significantly higher in ZX978 both under non-stress and drought-stress conditions over ND476 (Fig 7C). The lower values of MDA in ND476 may suggest that at cellular level, this genotype is better equipped with efficient free radical quenching system than ZX976, which offers ND476 comparably better protection against oxidative stress. Overall, these results revealed that 12-days drought treatment affected physiological changes of two cultivars, as well as indicated that ND476 has better drought tolerance than ZX978.

### Overlapping DEGs related to 'response to stimuli' between ND476 and ZX978 under drought conditions

Maize is relatively sensitive to drought stress, thus, in this research, we observed some common characteristics between the tolerant hybrid line ND476 and the sensitive hybrid line ZX978 under drought stress conditions. Several DEGs shared by the two cultivars were annotated to 'response to stimuli' under BP category, including abscisic acid (ABA), peroxidase, glutathion S-transferase (GST), HSPs, dehydrins (DHNs) and TFs among others (S8 Table). The phytohormone ABA plays key roles in multiple aspects of plants response to abiotic stress. Under drought conditions, several ABA related genes are synthesized, ABA combines with receptor proteins to control the opening and closing of the stomata as well as water exchange, which then triggers stress responsive genes and modulation of the antioxidant defense system to protect plants from reactive oxygen species (ROS) induced damage [32,33]. Bano et al [34] reported that ABA induces and increases antioxidant enzyme activities. Here, ROS scavenging enzymes POD and GST have been identified. Peroxidases located in the vacuole and the cell wall act as the first line of cell defense by detoxifying ROS-generated hydrogen peroxide [35]; peroxidase roles in stress response have been extensively discussed in our previous research [36]. GST is a key cellular detoxification enzyme playing a critical role under water-deficit conditions. In line with our study, GSTs exhibited increased abundance in wheat [37], rice [38] and barley [39] reacting to osmotic stress.

Drought response genes such as DHNs, a group of the LEA proteins, play critical roles in preventing intracellular water loss [40]. The expression of DHNs has been detected in both tolerant and sensitive maize lines in response to drought stress [41]. Previously, several LEA proteins related to cellular dehydration have been cloned under water-deficit conditions. Barley LEA gene *HVA1* over-expressed in transgenic rice improved the performance of transgenic rice by offering cell membrane protection under drought stress conditions [42]. Water-deficit stress triggered the constitutive expression of HSPs, which then assisted in proper folding or unfolding of proteins against denaturation, thereby contributing to cellular homeostasis under drought stress conditions [43]. In our previous study, the expression of HSPs was also crucial in maize drought response at the seedling stage [22]. Taken together, our results showed that under drought stress conditions, both drought-tolerant and drought-sensitive hybrid lines enhanced their cellular redox homeostasis and induced drought response genes in order to regulate the steady state level of ROS and resist drought stress.

### TF related genes are an essential component of plants response to drought

TFs play an important role in regulating drought stress, participating as essential controllers of numerous downstream stress responsive genes [44]. A large number of TFs were observed to be differentially expressed in two hybrid lines including C_2_H_2_, AP2/EREBP, MYB-related, bHLH, MYB, WRKY, bZIP, and NAC (Table 1). The roles of TFs in abiotic stress tolerance in various crops including maize are well-documented [38]. Previously, in a transcriptome study to detect drought-induced responsive genes in flowering maize plants, a subset of drought-responsive TFs were identified including C_2_H_2_, NAC and bHLH to be prominent [45]. Zhang et al. [20] found numerous TF genes commonly shared between two drought-contrasting maize inbred lines under field drought treatment, including one AP2/EREBP, five bZIP, and three MYB. Furthermore, in a study by Thirunavukkarasu et al [46] identified several drought responsive TFs such as WRKY, C_2_H_2_, MYB, bHLH, bZIP and NAC to be differentially expressed in both tolerant and sensitive maize lines in response to water-deficit stress. In short, this discussion fortifies the essential role TFs play in regulating drought response in maize, with various TF families exhibiting differential responses and interacting with each other in complex gene regulatory networks.

### Photosynthesis related genes were differentially altered under drought stress

Photosynthesis is one of the critical processes to be influenced by drought, via reduced CO_2_ diffusion to the chloroplasts and metabolic restrictions. In the current investigation, our KEGG and GO enrichment analyses showed that photosynthesis was dominating in ZX978at the V12 stage and ND476 at the VT stage. We then paid particular attention to the regulation of photosynthesis related genes in response to drought stress of those two stages (Figs 2 and 3B). A total of 41 photosynthesis related genes were differentially expressed in ZX978 at the V12 stage. Among these, 40 genes displayed decreased transcript abundance, including nine (*PsbQ-like1*, *LPA19*, two *PsbQ-like 2*, *PsbQ (B)*, *CP-47*, *PsbQ (A)*, *PsbP*, and *PSB28*) encoding different protein subunits of the photosystem II, two genes (*LHCA6* and *LHCA5*) encoding subunits of the light-harvesting chlorophyll-protein (LHC) complex, and four genes encoding subunit NDH-N of NAD(P)H. Only one RuBisCO activase gene displayed increased abundance in ZX978 at the V12 stage (S9 Table).

A total of nine photosynthesis related genes were identified in ND476 at the VT stage. Among these, eight showed down-regulated expression after drought treatment, including three (*PSI-D*, *PSI-N*, and *PSI-G*) encoding different protein subunits of the photosystem I, one gene (*PsbQ*) encoding different protein subunits of the photosystem II. Only one gene, FAD/NAD(P)-binding oxidoreductase, involved in oxidoreductase activity, was up-regulated in response to drought treatment (S9 Table). The same photosynthesis related genes were exhibited decreased expression in maize under drought stress [41]. In a study by Thirunavukkarasu et al [46], genes involved in photosynthesis were reported to be down-regulated in maize drought stress response. Taken together, the expression profiles of the photosynthesis related genes showed that photosynthesis efficiency was inhibited by drought stress and that stress response was cultivar-specific as well as growth stage-specific.

### Significantly enriched metabolic pathways of DEGs under drought stress

Multiple metabolic processes are affected by drought stress. Here, our comparative analysis of drought-tolerant ND476 and drought-sensitive ZX978 maize hybrid lines revealed some notable similarities and differences in terms of metabolism pathways enriched in response to drought stress. Specifically, our KEGG pathway enrichment analysis revealed that ribosome and photosynthesis pathways were significantly enriched in both cultivars after drought treatment (Fig 2). Ribosome is the site for protein synthesis, one of the basic biological processes affected by abiotic stress [47], hence the pathway was significantly enriched. To adapt to water-deprived conditions, plants would immediately close the stomata, thereby reducing the leaf gas exchange. This would negatively influence photosynthetic parameters [48]. Previously, Zhao et al [49] observed photosynthesis pathway to be significantly enriched in maize in response to water-deficit stress.

In the tolerant hybrid line ND476, 'starch and sucrose metabolism’ pathway was highly enriched in response to drought stress at the VT stage (Fig 2A). The importance of starch and sucrose metabolism pathway in water-deficit stress response in maize is well-documented [36]. Starch and sucrose metabolism plays important roles in cellular energy provision, thereby contributing to plants tolerance to stressful conditions. Phenylpropanoid biosynthesis and metabolism pathways were the most significantly enriched in ND476 at the R2 and R4 stages under drought stress conditions (Fig 2A). Phenylpropanoid metabolism is the first step in secondary metabolites (flavonoids, phenylpropanoids, etc.) biosynthesis, which are subsequently activated to effect stress tolerance [47]. Previously, Li et al [50] observed phenylpropanoid metabolism pathway to be significantly enriched in *Bothriochloa ischaemum* L. drought stress response.

Contrastingly, PGI and MAPK signaling pathways were the top most significantly enriched in the sensitive hybrid line ZX978 at the VT stage after drought treatment (Fig 2B). Similarly, in our previous report, PGI pathway was significantly enriched in drought sensitive wide-type Vp16 maize line in response to osmotic stress [51]. Further, the PGI pathway has been reported to be significantly enriched in cotton under drought stress [52]. Interestingly, the MAPK signaling pathway was also significantly enriched in ZX978 at the R2 stage, indicating that it plays a critical role in maize response to drought (Fig 2B). The signal transduction via MAPK cascade offers a rapid amplification and relaying of external stimuli via activation or de-activation of enzymes through phosphorylation/ de-phosphorylation [53].

Moreover, plant hormone signal transduction pathway has been significantly enriched under drought conditions in ZX978 at the R2 stage. Shinde et al [47] and Serrano et al [54] revealed that plant hormone signal transduction pathway takes part in abiotic stress adaptation by ubiquitin-mediated proteolysis or ABA-mediated response. As for the R4 stage, sulfur metabolism pathway was significantly enriched in the sensitive genotype ZX978 in response to drought stress (Fig 2B). A review by Chan et al [55] provides an adequate summary of the significant roles sulfur metabolism play in drought stress signaling and response. Taken collectively, the significant difference in drought tolerance between the two hybrid genotypes could be emanating from the difference in the number and specific types of genes enriched in the common/shared, as well as the different metabolic pathways.

### Specific expression patterns of the drought-responsive genes

To better understand the regulation patterns of genes identified at each drought treatment stage, K-means approach was employed to cluster those DEGs into clear and distinct expression profiles in both ND476 and ZX978 hybrid lines. We observed that most special clusters were related to drought defense response (Fig 3B and 3C). Those clusters offered ample evidence of the involvement of DEGs in molecular modulation of drought stress response at different growth stages in the two maize genotypes. Here, 116 DEGs grouped in cluster 6 and enriched for defense response related functions showed significantly high expression after drought treatment in ND476 at the V12 stage (Fig 3A and 3B). We observed that some of those genes (including HSPs, bHLH, ARF-transcription factor, peroxidase and pathogenesis-related (PR) protein) were also annotated to ‘response to stimuli’ (S3 Table). PR proteins have been implicated in plant development and defense response processes in response to abiotic stress [56].

In the drought tolerant line ND476 at the VT stage, we identified 57 stage-specific DEGs in cluster 4 were involved in photosynthesis and showed high expression, whereas 48 DEGs in cluster 5 were related to response to stress and exhibited low expression under both well-watered and drought treatment conditions. Among these, peroxidase, trehalose-6-phosphate phosphatase 8, MYB, bZIP, glyceraldehyde-3-phosphate dehydrogenase, photosystem II core complex protein PsbY and photosystem I subunit d1, were mainly down-regulated in cluster 4 (S3 Table). However, the DEGs in cluster 5 including protein phosphatase 2C (PP2C), protein phosphatase homolog, HSPs, GST and lysine-ketoglutarate reductase were up-regulated in response to drought stress (S3 Table). Protein kinases (PKs) are sensor-responder genes which initiate phosphorylation cascades and play essential roles in water-deficit responses [57]. A large number of signaling genes were part of the cluster 1 at the R2 stage. The predominant members of this cluster were receptors (ABA receptor PYL5, zinc, glutamate, ABC receptor, etc.), non-specific lipid-transfer protein, Ca^2+^-dependent phospholipid-binding protein family and LRR receptor-like serine/threonine-protein kinases (S3 Table). As for the R4 stage, cluster 4 comprised 76 stage-specific genes associated with transport and exhibiting high expression under both well-watered and water-deficit treatment conditions. The principal genes of cluster 4 were transporters (oligopeptide, high affinity nitrate, sulfate, cationic amino acid and GDP-mannose) (S3 Table). At the initial stages of abiotic stress, stress signal receptors, transductors and transporters on cell membranes perceive stress and transmit the stress signals to the target genes, triggering subsequent plant physiological responses [58].

Although the photosynthesis-related genes of cluster 4 were down-regulated in ND476 at the VT stage, they were greatly down-regulated in cluster 2 in ZX978 at the V12 stage post drought treatment. Moreover, the main components of this cluster were proteins of eukaryotic small subunit ribosomal RNA families (S4 Table). Interestingly, these photosynthesis related genes also exhibited high expression in clusters 5 and 6 at the VT stage post drought treatment. We also found out that the protein kinase related genes (calcium-dependent protein kinase and receptor-like kinase), defense response genes (PRs and GST) and TFs (AP2-EREBP and bZIP) in cluster 5, as well as cellular redox homeostasis related enzymes (peroxidases) and defense genes (PRs) in cluster 6 had increased abundance after drought treatment (S4 Table).

More remarkably, the 'response to abiotic stimulus' and 'response to stress' related DEGs in cluster 7, including HSPs, DHNs, PRs, AP2-EREBP and WRKY, showed high expression under drought conditions at the VT stage, but low expression at the V12, R2 and R4 stages (S4 Table). A large number of researches have reported these stress responsive genes to play vital roles in maize drought stress response [21,22]. Meanwhile, as for the R2 stage, plant hormone signaling related genes in cluster 4, including brassionosteroid receptors (BRs) and ABA; NAC-transcription; and stress-induced protein, PRs were down-regulated. However, cluster 5 DEGs, including PP2C, DHNs, bHLH and LEA proteins were up-regulated in response to drought stress (S4 Table). PP2C has been reported to play key roles in multiple ABA-activated signal transduction processes including stress acclimation [59]. Both cluster 4 and cluster 6 DEGs, enriched for defense-response and response-to-stress functions, showed significantly high expression under well-watered conditions, but low expression after water-limited conditions at the R4 stage. These results showed that drought responsive genes exhibited different expression patterns between the two maize genotypes and across different growth stages, and even within a single cultivar.

### Quantitative RT-PCR validation of the result of RNA-seq

Quantitative RT-PCR (qRT-PCR) analysis is the most commonly used method to validate the accuracy of the RNA seq transcriptome data. Benefits of qRT-PCR over conventional methods for measuring RNA include its specificity, high sensitivity, well reproducibility, wide dynamic quantification range, and high-throughput capacity [60]. To experimentally validate the results of transcriptome sequencing, we further performed qRT-PCR analysis on twenty randomly selected genes. Resultantly, all the tested genes showed similar expression patterns to the results from RNA-seq (Fig 6). In addition, correlation analysis between qRT-PCR and RNA-seq showed that qRT-PCR and RNA-seq results were highly correlated (R^2^ = 91.55%) (S6 Fig). Thus, qRT-PCR confirmed the accuracy of our RNA-seq data.

### Proposed hypothetical model for maize drought stress response

**Fig 8:**
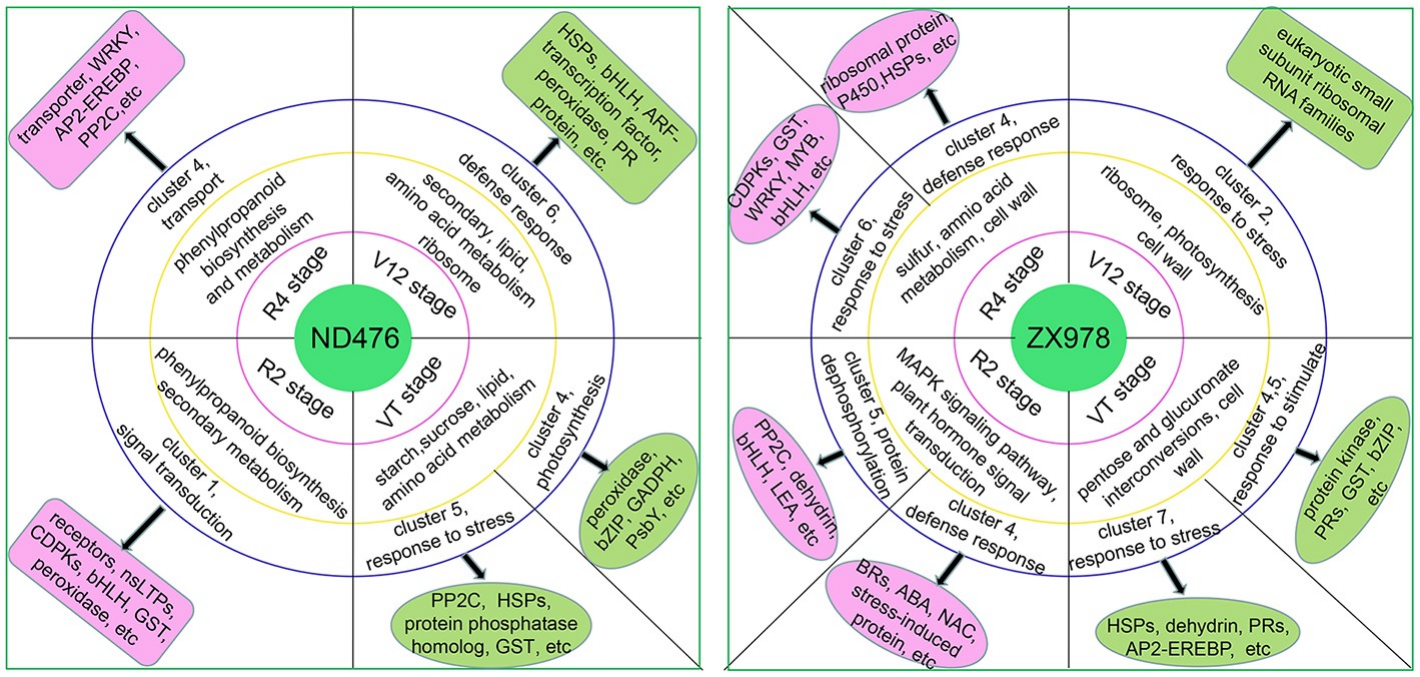
Molecular models of drought tolerance in maize tolerant hybrid line ND476 and sensitive hybrid line ZX978.

We propose a hypothetical model for maize drought stress response of two hybrid cultivars at four growth stages based on the transcriptome-level analysis (Fig 8). The key genes screened in this study may provide a foundational basis for our future targeted cloning studies.

## Conclusions

In the present study, we have performed a comparative leaf transcriptome analysis of two maize hybrid cultivars contrasting in drought tolerance exposed to 12 days drought treatment at V12, VT, R2 and R4 stages. Based on an RNA-seq approach, a total of 3451 and 4088 DEGs were identified from drought-tolerant ND476 and drought-sensitive ZX978 from the four experimental comparisons. Changes in these genes effected corresponding metabolic pathway responses related to drought tolerance in maize. Our results showed that maize drought stress adaptation is a cultivar-specific and a stage-specific response process. In drought-tolerant ND476, the DEGs associated with the ribosome, starch and sucrose metabolism, phenylpropanoid biosynthesis and phenylpropanoid metabolism pathways were predominantly at the V12, VT, R2 and R4 stages, respectively. However, in drought-sensitive ZX978, DEGs related to ribosome, PGI, MAPK signaling and sulfur metabolism pathways were dominant at the four respective stages. Meanwhile, K-means analysis clustered those DEGs of ND476 and ZX978 into clear and distinct expression profiles at each stage. Several functional and regulatory genes were identified in the special clusters related to drought defense response. Additionally, physiological analysis results corroborated our RNA-seq transcriptome findings. Overall, our findings may help in clarifying the important cultivar-specific and growth-stage-specific molecular mechanisms regulating maize drought stress responses. More crucially, the key genes and metabolic pathways identified here could serve as valuable genetic resources or selection targets for our future targeted cloning and downstream analysis studies.

## Materials and methods

### Plant materials and drought treatment

Two maize hybrid cultivars contrasting in drought tolerance (drought tolerant ND476 and drought-sensitive ZX978) were used in this study Maize hybrid line ND476 is a comparably drought-tolerant cultivar developed by the Dryland Research Institute of Hebei Academy of Agricultural and Forestry Sciences (Hengshui, China) and ZX978 is a comparably drought sensitive line bred by the Hebei Zhongxin Seed Technology Company Limited (Handan, China). Both ND476 and ZX978 hybrid cultivars were identified as drought- tolerant and sensitive, respectively, through laboratory and field screening of numerous maize lines for drought tolerance by our lab (North China Key Laboratory for Crop Germplasm Resources of the Education Ministry, Hebei Agricultural University, Baoding, China, patent ID: 201910634005.2). The seeds used in this experiment were provided by our lab. The seeds were sown in a fully automated rain-proof shelter at Qing Yuan Experimental Station, Baoding, China (115.5602790 E; 38.7950930 N; 118 m) in 2018. No specific permissions were required for use of Qing Yuan Experimental Station, since it belong to the Department of Crop Genetics and Breeding, College of Agronomy, Hebei Agricultural University, Baoding, China, whose Head is the corresponding author, Huijun Duan (H.D). The authors further confirm that the field studies did not involve endangered or protected species. The canopy of the shelter was normally opened, and could be closed automatically on rainy days to enable continuous drought treatment. Plants were grown in experimental plots of 25 m^2^ (5m × 5m) each, with 60 cm * 30 cm plant spacings. The soil water content of control groups was kept between 70–80%, while the drought treatment groups was kept between 15–20%, monitored using a TZS-1 soil moisture measurement instrument (Zhejiang Tuopu Technology Co. Ltd, Zhejiang, China) [61]. The field arrangement followed a randomized complete block design, with the well-watered and water-deprived groups replicated three times. To prevent the transverse infiltration of soil moisture, building waterproof membranes of one-meter depth were put between control and treatment plots.

**Fig 9:**
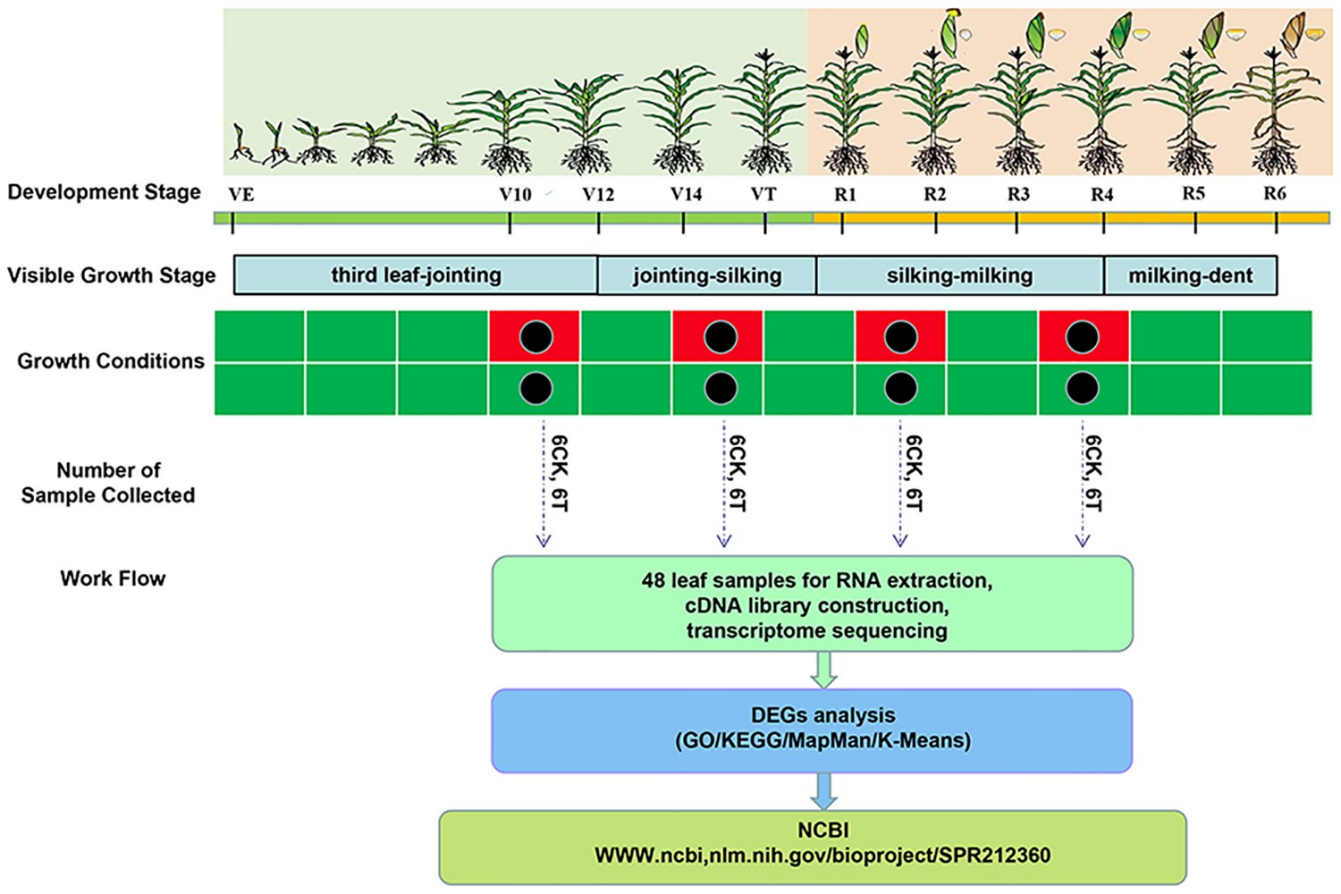
Schematic representation of the leaf transcriptome sequencing experiment under control and drought stress treatment in ND476 and ZX978 maize hybrid cultivars from vegetative to reproductive growth stages. Black dots show the time of leaf sample collection after 12 days of treatment exposure.

According to the maize growth cycle, water-deficit treatment was instituted at four different growth stages. Two maize hybrid cultivars were water deprived (a) from eight fully-expanded-leaf (FEL) to twelve FEL (V12) period, (flared stage); (b) from fourteen FEL until the tassel was visible (VT) (tasseling stage); (c) from self-pollination until 12 days post pollination (DPP), that is, the prophase of grain filling stage (R2); and (d) from 13 DPP until 24 DPP, that is, the anaphase of the grain filling stage (R4) (Fig 9). For each growth stage after twelve days drought stress exposure, leaf tissues of three biological replicates were collected from both the control and drought-stressed ND476 and ZX978 genotypes (48 samples in total). All the flag leaves were then immediately frozen in liquid nitrogen and stored at -80°C for further analysis.

### Physiological parameter measurement

Leaf samples for RWC, antioxidant enzyme activity assays and lipid peroxidation analysis for both ND476 and ZX978, from both control and drought treatment conditions, were collected at the start and different intervals (days) of a 12-day drought treatment period. Leaf RWC was determined according to Galmés et al. [62]. Guaiacol peroxidase (POD) enzyme activity was assayed at 470 nm by Han`s guaiacol method [63]. The measurement of lipid peroxidation, which is determined by measuring malondialdehyde (MDA), was assessed with the thiobarbituric acid reaction as previously described [64]. POD activity expressed as U.g fresh weight (FW)^-1^. MDA content expressed as nmol .g FW^-1^. Each physiological parameter measurement on each maize cultivar under each growth condition was replicated three times using independent but parallel approaches.

### RNA extraction, cDNA library construction and Illumina sequencing

Total RNA was isolated from the flag leaf tissues of the two hybrid lines (ND476 and ZX978) at four different (V12, VT, R2, R4) growth periods and two treatment (water sufficient, control; and water deficit, drought) conditions using Trizol reagent (Invitrogen, Carlsbad, CA, USA). Three independent biological samples were obtained giving 48 samples in total. RNA was treated with DNase I (QIAGEN, Pudong, Shanghai, China) to eliminate contaminating genomic DNA. RNA degradation and contamination (integrity) were monitored on 1% agarose gel. RNA quality was determined by 2100 Bioanalyser (Agilent) and quantified using the ND-2000 (NanoDrop Technologies Inc., Wilmington, DE, USA). Only RNA samples with OD260/280 ratios from 1.8 to 2.2, OD260/230 ratio≥2.0, RIN (RNA integrity number) ≥6.5, and 28S:18S≥1.0 were used for downstream analysis. The cDNA libraries for Illumina sequencing were prepared following TruSeqTM RNA sample preparation Kit from Illumina (San Diego, CA, USA) using 1μg of total RNA following the manufacturer`s protocols. Then, the synthesized cDNA were purified and resolved with fragmentation buffer for end reparation and single nucleotide A (adenine) addition. After PCR amplification, TBS380 (Turner Biosystems, USA) was used in quantification and qualification of the sample library. Finally, cDNA library sequencing was performed at Shanghai Major Bio-pharm Biotechnology Co., Ltd. (Shanghai, China) using Illumina Novaseq 6000 (2×150bp read length).

### Sequencing reads processing, mapping and gene expression quantification

Raw data (raw reads) generated by the Illumina Novaseq 6000 system were initially processed and quality trimmed by SeqPrep (https://github.com/jstjohn/SeqPrep) and Sickle (https://github.com/najoshi/sickle) with default parameters. In this step, clean data (clean reads) were obtained by removing reads with≥ 2 mismatches in adapter, reads containing ploy-N, and reads with more than 2 or 5 bases having score ≤ 3 in the first 15 or 25 bases from raw data. Then, all high quality clean reads were separately aligned to reference genome (ZmB73_Ref-Gen_v4) using TopHat (http://tophat.cbcb.umd.edu/, version2.1.1) software [65]. The aligned reads were filtered using a series of rules: sequencing reads should be uniquely matched to the genome allowing up to 2 mismatches, without insertions or deletions. All the downstream analyses were based on those reads with a perfect match or one mismatch to the reference data. The FPKM of each gene was calculated based on the length of the gene and reads count mapped to that gene. Finally, the FPKM values > 1 were used to determine genes expressed.

### Homology search and functional annotation

Gene functions were annotated against the following databases: Nr (NCBI non-redundant protein sequences) (https://www.ncbi.nlm.nih.gov/), Swiss-port (a manually annotated and reviewed protein sequence database) (https://web.expasy.org/docs/swiss-prot), COG (Clusters of Orthologous Groups) (https://www.ncbi.nlm.nih. gov/ COG/), KEGG (Kyoto Encyclopedia of Genes and Genomes) (http://www.genome.jp/kegg) and GO (Gene ontology) (http://www.geneontology.org) using BLASTX search. The threshold E-value was set to 1E-5 and a 70% query coverage threshold was used to discard partial /single-domain protein matches.

### Differentially expressed genes (DEGs) detection and function enrichment analysis

We performed DEGs analysis for both drought-stressed and control conditions from the V12 stage to the R4 stage for ND476 and ZX978 genotypes in order to characterize transcriptional variations that occur in response to water-deprivation. Differential expression analysis of two conditions/groups was conducted using the EdgeR R package (Empirical analysis of Digital Gene Expression in R, http://www.bioconductor.org/packages/2.12/bioc/html/edgeR.html) (S10 Table) [66]. DEGs were determined using p-value < 0.05 and fold change ≥ 1.5 [67,68]. KEGG significant enrichment analysis to identify the key pathways (FDR < 0.05) was carried out by KOBAS 2.1.1 (http://kobas.cbi.pku.edu.cn/download.php) [69]. Additionally, MapMan 3.5.1R2 was used to visualize metabolism processes related DEGs at the whole drought period of two hybrid cultivars. The similar expression patterns of the DEGs were identified by K-means clustering on the log transformed fold induction expression values using R package. The Q-value cutoff (clusterCutoff) for the K-means clustering was determined automatically using the default settings and set at < 0.5. For each cluster, GO enrichment analysis was performed for the clustered DEGs using the singular enrichment analysis (SEA) function of the agriGO web-based program (http://systemsbiology.cau.edu.cn/agriGOv2/index.php) [70]. Multiple testing corrections were conducted to filter overrepresented biological process (BP) terms by using FDR controlled at 5%.

### Identification of key drought responsive–, cultivar-specific and stage-specific genes and metabolic pathways

The expression abundances of each gene appearing in the two libraries (control versus drought treatment) were used to determine the expression changes of the gene in response to drought stress. Then, the total DEGs observed to respond to drought stress at each crop growth stage (those that fell within the selection criterion specified in the previous section) were analyzed by way of Venn diagram analysis to identify those DEGs that were only expressed at a particular growth stage excluding others (growth stage specific) and in a particular genotype excluding the other (cultivar-specific). After filtering, the important drought responsive genes were specified by meeting the following criteria: the DEGs that specifically expressed in the tolerant genotype ND476 after drought treatment; DEGs shared between the drought-sensitive and drought-tolerant hybrids after drought treatment (SD_TD); DEGs of the tolerant cultivar that were also differentially expressed in SD_TD; and the common DEGs shared by the two hybrids under drought stress (TC_TD and SC_SD). This was supported by revealing their functional annotation and roles through GO and KEGG analyses, as well as refereeing to previously published works [71].

### Quantitative real Time-PCR (qRT-PCR) analysis

To validate gene expression levels detected by Illumina RNA-seq, quantitative real-time PCR (qRT-PCR) was performed using a C1000 (CFX96 Real-Time System) Thermal Cycler (Bio-Rad, Hercules, CA, USA). RNA for qRT-PCR analysis was isolated independently from that isolated for RNA-seq; RNA was procedurally extracted as already described above. Three biological sample replicates were used for analysis. For cDNA synthesis, 1μg of total RNA of the samples was reverse-transcribed in a total volume of 20 μL, using HiFiscript cDNA Synthesis Kit (CWBIO, Beijing, China). Real time quantitative PCR was carried out using 2×Fast Super EvaGreen ^®^ qPCR Mastermix (US Everbright Inc., Suzhou, China) in a Bio-Rad iQ5 thermo cycler (Bio-Rad, Hercules, CA, USA). A total of 20 DEGs were randomly chosen and gene-specific primers for each DEG were designed using Primer Premier 5 Designer (Premier Biosoft International, Palo Alto, CA, USA) (S11 Table). A steady and consecutive expressed maize gene GAPDH (accession no. X07156) was used as the internal control to normalize the other DEGs results. Additionally, a negative control was added. The qRT-PCR program was performed with 1 μl of template cDNA, 1 μl of forward primer (50 pmol), 1 μl of reverse primer (50 pmol), 10 μl of SYBR Green mix (US Everbright Inc., Suzhou, China) and 7μl ddH2O in a total reaction volume of 20 μl. Each sample had three technical replicates. The relative mRNA abundance was determined by a two-side Student's t-test [72].

### Statistical analysis of the physiological changes

A general mean ± standard error across each repeated measurement was calculated and used. The significance of the differences between the control and drought treatments were determined with two-way ANOVA and least significant difference tests (*p* < 0.05) using SPSS v. 22.0 statistical package (SPSS Institute Ltd., Armonk, NY, USA).

## Supporting information

S1 Fig Intersample (PCA) analysis of forty-eight leaf tissue samples used for transcriptome sequencing. There are relative coordinate points on the principal component after the samples are analyzed by dimension reduction. The closer the distance between two points, the higher the similarity between the samples. (A) The leaf samples of drought-tolerant hybrid line ND476. (B) The leaf samples of drought-sensitive hybrid line ZX978.
(DOCX)

S2 Fig Gene ontology annotation analysis of the differentially expressed genes (DEGs) identified in four experimental stages of ND476 and ZX978. The GO analyses results here shown the GO terms from biological processes (BP) categories combined (A) GO annotation of DEGs identified in ND476 four experimental stages; (B) GO annotation of DEGs identified in ZX978 four experimental stages.
(DOCX)

S3 Fig Cluster analysis of DEGs identified during drought treatments in ND476. Heat map illustrating the expression profiles of the DEGs of ND476 at (A) VT stage, (B) R2 stage, and (C) R4 stage for both well-watered (control, C) and drought treatment (T) conditions. The bars on the left side represent the hierarchical clustering analysis results while the clusters on the right side show the analysis results of the gene expression profiles with the K-means algorithm.
(DOCX)

S4 Fig Cluster analysis of DEGs identified during drought treatments in ZX978. (A) Heat map illustrating the expression profiles of the DEGs of ZX978 at V12 stage, (B)VT stage, (C) R2 stage, and (D) R4 stage both of well-watered and drought treatments. The bars on the left side of represent the different clusters, while the results of the cluster analysis of the gene expression profiles with the K-means algorithm are presented on the right side.
(DOCX)

S5 Fig Overview of metabolic responses to drought. (A) The DEGs of ND476 identified at VT stage, (B) the DEGs of ZX978 identified at VT stage, (C) the DEGs of ND476 identified at R2 stage, (D) the DEGs of ZX978 identified at R2 stage, (E.) the DEGs of ND476 identified at R4 stage, (F) the DEGs of ZX978 identified at R2 stage after drought treatment as visualized by Mapman.
(DOCX)

S6 Fig Validation of RNA-seq expression data by qRT-PCR analysis. Validation was performed using 10 randomly selected DEGs in (A) ND476, and (B) ZX978, respectively. The plots demonstrate the expression ratio in log scale with base of two. The X-axis indicates qRT-PCR log scale, the Y-axis indicates RNA-seq log scale.
(DOCX)

S1 Table Summary of RNA sequencing results for the forty-eight maize leaf samples. (XLS)

S2 Table The Spearman correlation of different samples based on FPKM values. (XLS)

S3 Table The DEGs classified into special clusters of ND476 four growth stages. (XLS)

S4 Table The DEGs classified into special clusters of ZX978 four growth stages. (XLS)

S5 Table The DEGs annotated to metabolism processes visualized by Mapman of ND476 four growth stages. (XLS)

S6 Table The DEGs annotated to metabolism processes visualized by Mapman of ZX978 four growth stages. (XLS)

S7 Table RNA-seq versus qRT-PCR expression values/data of the 20 representative DEGs. (XLS)

S8 Table The DEGs of the ND476 and ZX978 four growth stages that were enriched in GO term 'Response to Stimuli'. (XLS)

S9 Table The DEGs of the ND476 VT stage and ZX978 V12 stage that were enriched in 'photosynthesis'. (XLS)

S10 Table R code used for EdgeR. (R)

S11 Table Gene specific primers used for qRT-PCR analysis. (XLS)
