# Supplementary material for: Comparative transcriptomic analysis of contrasting hybrid cultivars reveal key drought-responsive genes and metabolic pathways regulating drought stress tolerance in maize at various stages
Source: PLoS One. 2020 Oct 15;15(10):e0240468. doi: 10.1371/journal.pone.0240468 (PMC7561095; doi:10.1371/journal.pone.0240468)
Supplement: S1 Fig — There are relative coordinate points on the principal component after the samples are analyzed by dimension reduction. The closer the distance between two points, the higher the similarity between the samples. (A) The leaf samples of drought-tolerant hybrid line ND476. (B) The leaf samples of drought-sensitive hybrid line ZX978. (DOCX) [file pone.0240468.s001.docx]

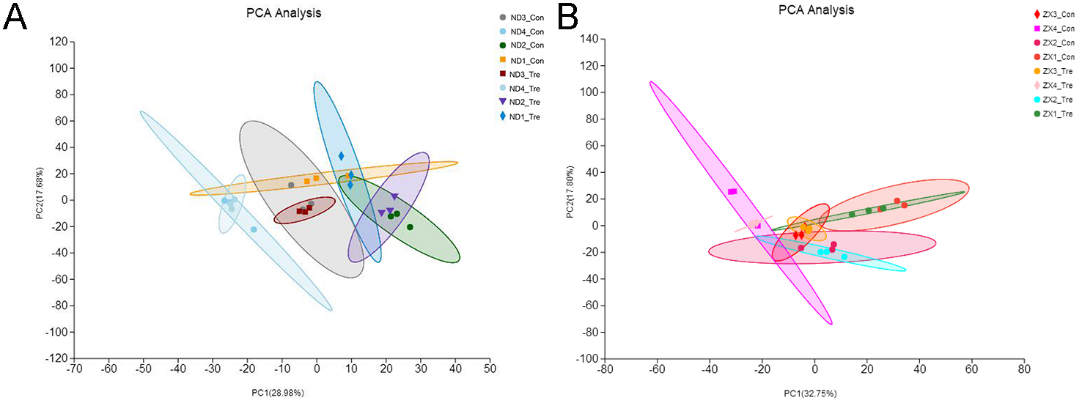


**S1 Fig.** **Intersample analysis of forty-eight leaf tissue samples used for transcriptome sequencing**. PCA analysis, there are relative coordinate points on the principal component after the samples are analyzed by dimension reduction. The distance of each sample point represents the distance of the sample. The closer the distance, the higher the similarity between the samples. (A) The leaf samples of drought-tolerant hybrid line ND476. (B) The leaf samples of drought-sensitive hybrid line ZX978.
