# Supplementary material for: Comparative transcriptomic analysis of contrasting hybrid cultivars reveal key drought-responsive genes and metabolic pathways regulating drought stress tolerance in maize at various stages
Source: PLoS One. 2020 Oct 15;15(10):e0240468. doi: 10.1371/journal.pone.0240468 (PMC7561095; doi:10.1371/journal.pone.0240468)
Supplement: S2 Fig — The GO analyses results here shown the GO terms from biological processes (BP) categories combined (A) GO annotation of DEGs identified in ND476 four experimental stages; (B) GO annotation of DEGs identified in ZX978 four experimental stages. (DOCX) [file pone.0240468.s002.docx]

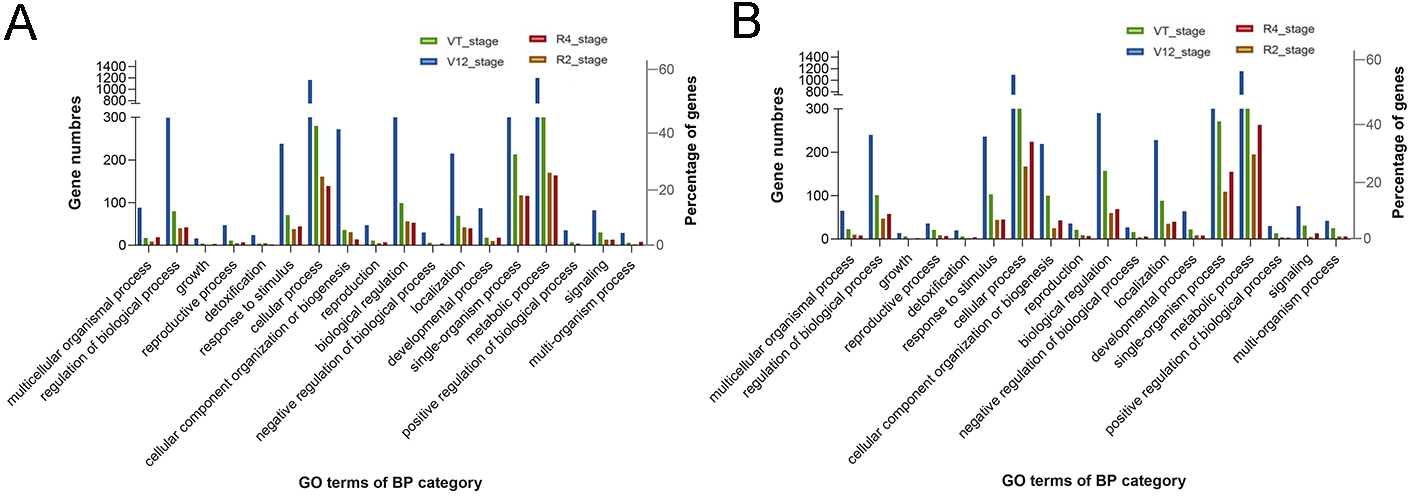


**S2 Fig. Gene ontology annotation analysis of the differentially expressed genes (DEGs) identified in four experimental stages of ND476 and ZX978.** The GO analyses results here shown the GO terms from biological processes (BP) categories combined (A) GO annotation of DEGs identified in ND476 four experimental stages; (B) GO annotation of DEGs identified in ZX978 four experimental stages.
