# Supplementary material for: Comparative transcriptomic analysis of contrasting hybrid cultivars reveal key drought-responsive genes and metabolic pathways regulating drought stress tolerance in maize at various stages
Source: PLoS One. 2020 Oct 15;15(10):e0240468. doi: 10.1371/journal.pone.0240468 (PMC7561095; doi:10.1371/journal.pone.0240468)
Supplement: S3 Fig — Heat map illustrating the expression profiles of the DEGs of ND476 at (A) VT stage, (B) R2 stage, and (C) R4 stage for both well-watered (control, C) and drought treatment (T) conditions. The bars on the left side represent the hierarchical clustering analysis results while the clusters on the right side show the analysis results of the gene expression profiles with the K-means algorithm. (DOCX) [file pone.0240468.s003.docx]

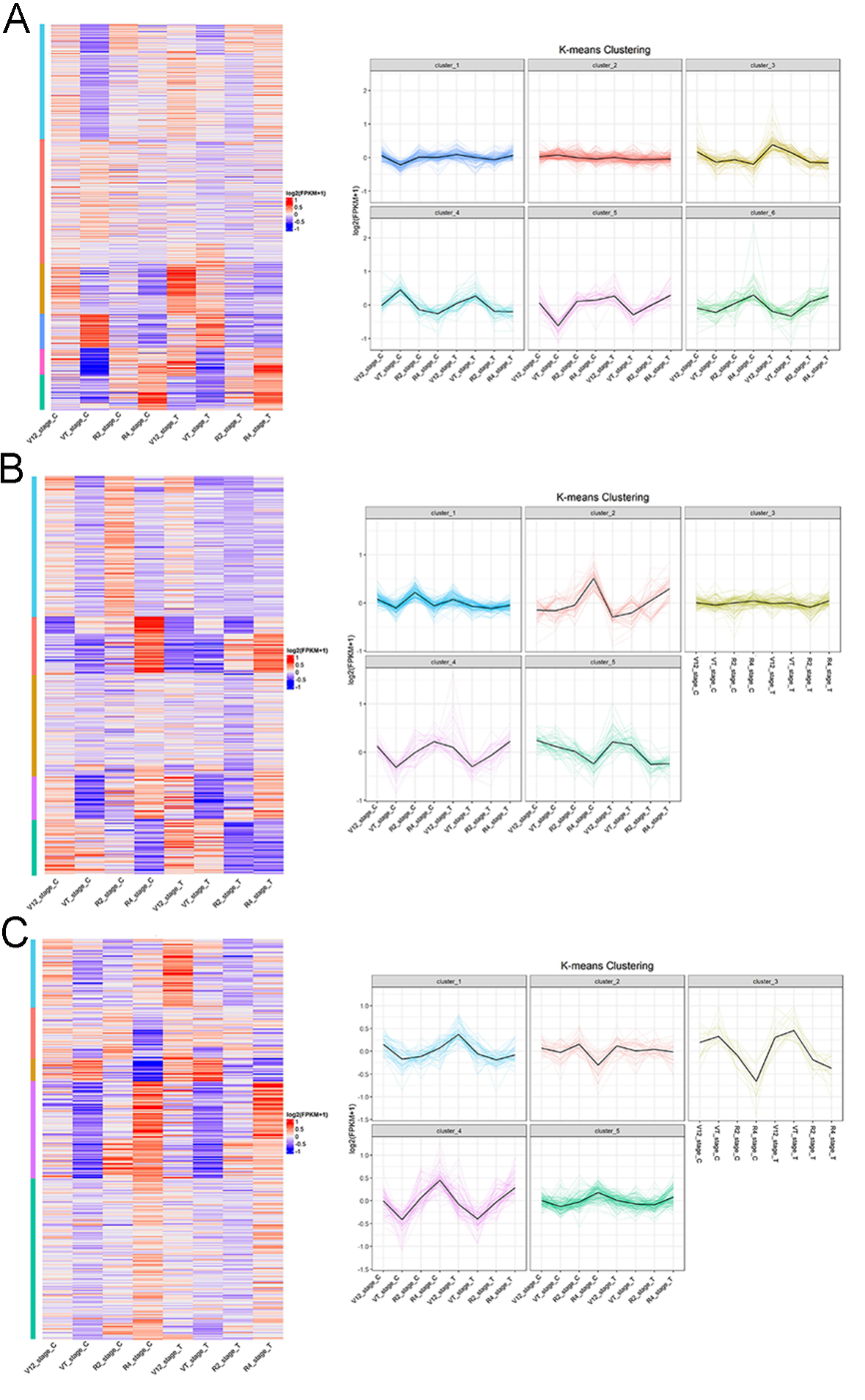


**S3 Fig. Cluster analysis of DEGs identified during drought treatments in ND476.** Heat map illustrating the expression profiles of the DEGs of ND476 at (A) VT stage, (B) R2 stage, and (C) R4 stage for both well-watered (control, C) and drought treatment (T) conditions. The bars on the left side represent the hierarchical clustering analysis results while the clusters on the right side show the analysis results of the gene expression profiles with the K-means algorithm.
