# Supplementary material for: Comparative transcriptomic analysis of contrasting hybrid cultivars reveal key drought-responsive genes and metabolic pathways regulating drought stress tolerance in maize at various stages
Source: PLoS One. 2020 Oct 15;15(10):e0240468. doi: 10.1371/journal.pone.0240468 (PMC7561095; doi:10.1371/journal.pone.0240468)
Supplement: S4 Fig — (A) Heat map illustrating the expression profiles of the DEGs of ZX978 at V12 stage, (B)VT stage, (C) R2 stage, and (D) R4 stage both of well-watered and drought treatments. The bars on the left side of represent the different clusters, while the results of the cluster analysis of the gene expression profiles with the K-means algorithm are presented on the right side. (DOCX) [file pone.0240468.s004.docx]

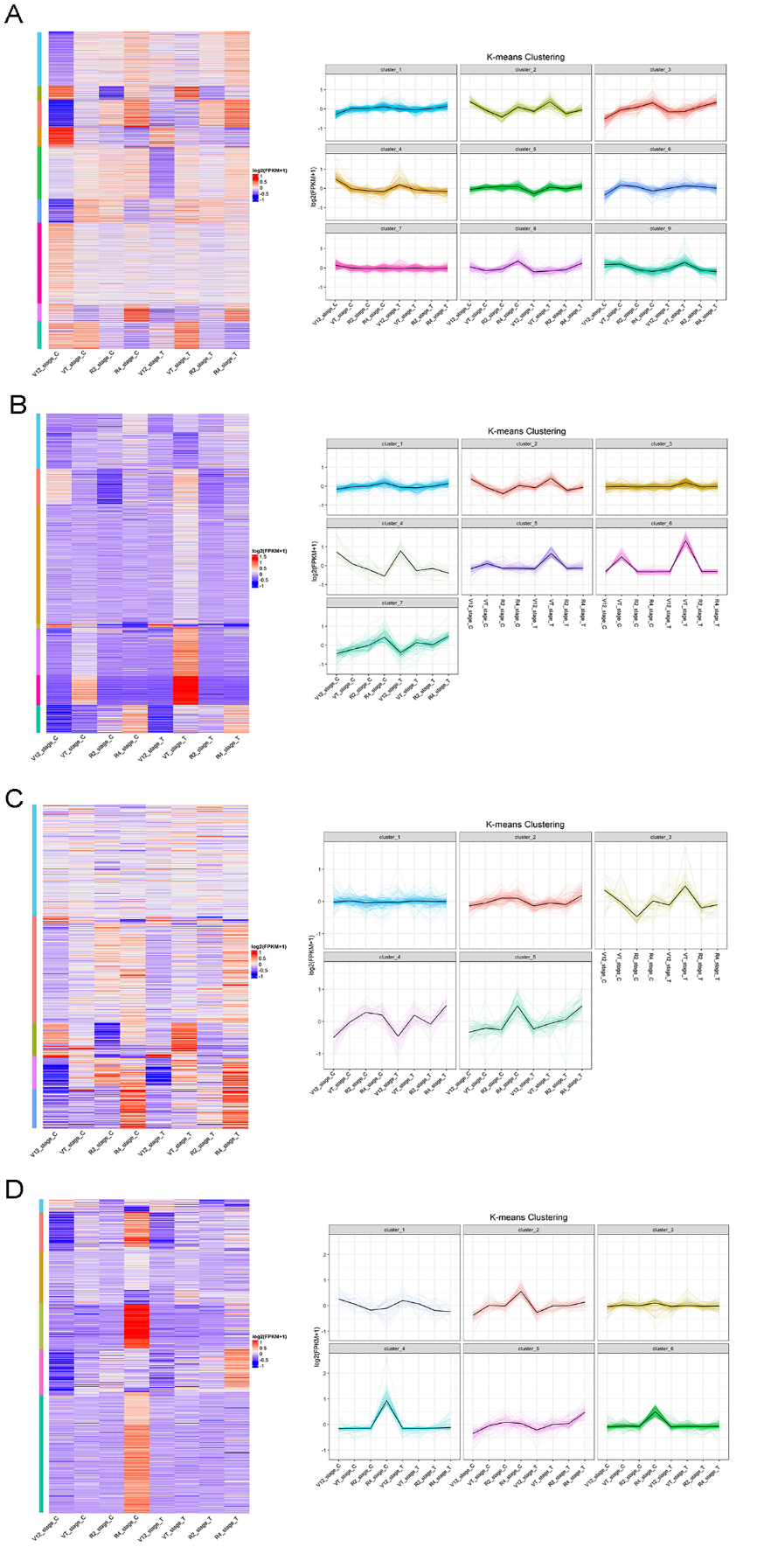


**S4 Fig. Cluster analysis of DEGs identified during drought treatments in ZX978.** (A) Heat map illustrating the expression profiles of the DEGs of ZX978 at V12 stage, (B)VT stage, (C) R2 stage, and (D) R4 stage both of well-watered and drought treatments. The bars on the left side of represent the different clusters, while the results of the cluster analysis of the gene expression profiles with the K-means algorithm are presented on the right side.
