# Supplementary material for: Comparative transcriptomic analysis of contrasting hybrid cultivars reveal key drought-responsive genes and metabolic pathways regulating drought stress tolerance in maize at various stages
Source: PLoS One. 2020 Oct 15;15(10):e0240468. doi: 10.1371/journal.pone.0240468 (PMC7561095; doi:10.1371/journal.pone.0240468)
Supplement: S5 Fig — (A) The DEGs of ND476 identified at VT stage, (B) the DEGs of ZX978 identified at VT stage, (C) the DEGs of ND476 identified at R2 stage, (D) the DEGs of ZX978 identified at R2 stage, (E.) the DEGs of ND476 identified at R4 stage, (F) the DEGs of ZX978 identified at R2 stage after drought treatment as visualized by Mapman. (DOCX) [file pone.0240468.s005.docx]

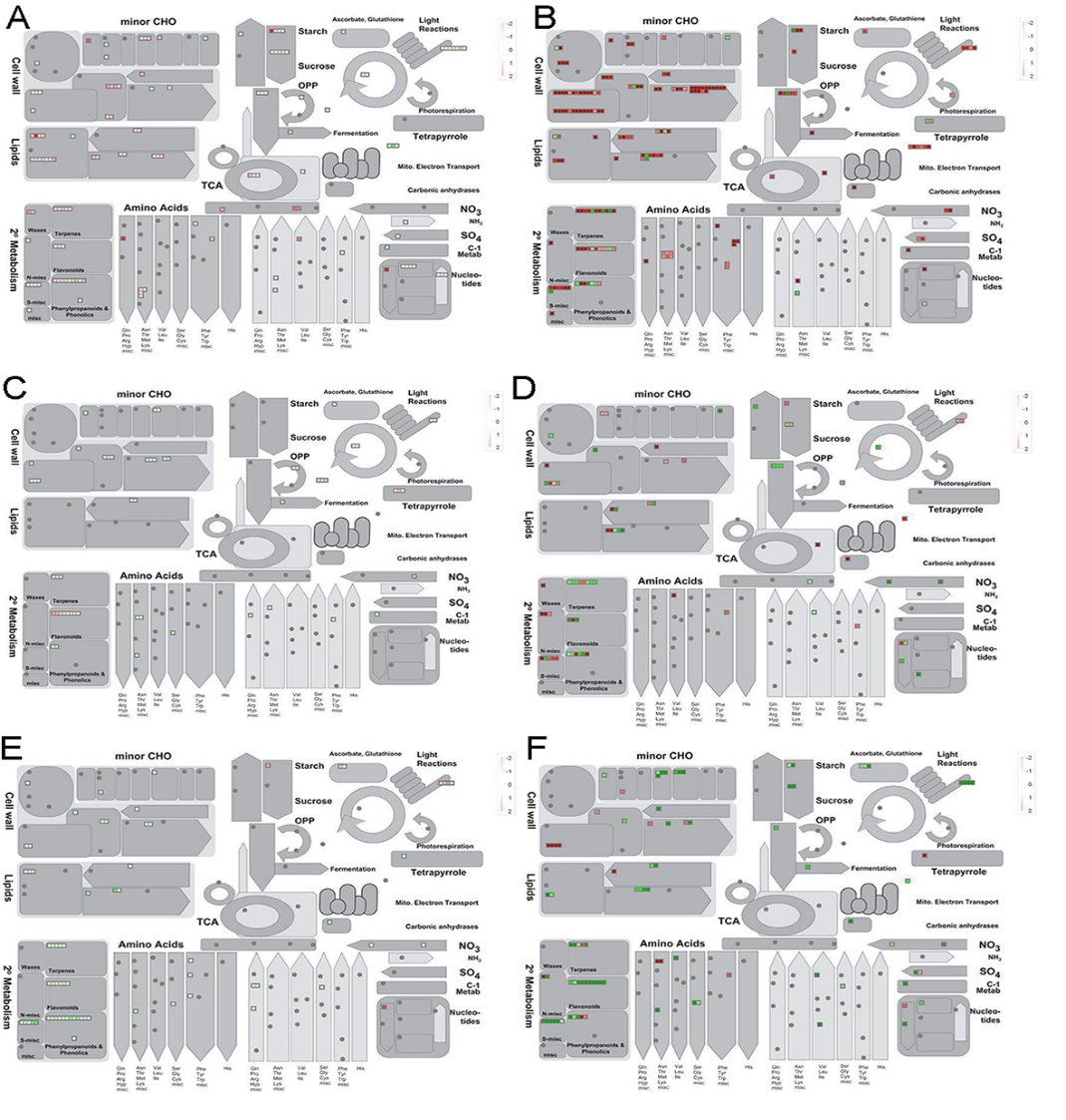


**S5 Fig. Overview of metabolic responses to drought.** (A) The DEGs of ND476 identified at VT stage, (B) the DEGs of ZX978 identified at VT stage, (C) the DEGs of ND476 identified at R2 stage, (D) the DEGs of ZX978 identified at R2 stage, (E) the DEGs of ND476 identified at R4 stage, (F) the DEGs of ZX978 identified at R2 stage after drought treatment as visualized by Mapman.
