# Supplementary material for: Comparative transcriptomic analysis of contrasting hybrid cultivars reveal key drought-responsive genes and metabolic pathways regulating drought stress tolerance in maize at various stages
Source: PLoS One. 2020 Oct 15;15(10):e0240468. doi: 10.1371/journal.pone.0240468 (PMC7561095; doi:10.1371/journal.pone.0240468)
Supplement: S6 Fig — Validation was performed using 10 randomly selected DEGs in (A) ND476, and (B) ZX978, respectively. The plots demonstrate the expression ratio in log scale with base of two. The X-axis indicates qRT-PCR log scale, the Y-axis indicates RNA-seq log scale. (DOCX) [file pone.0240468.s006.docx]

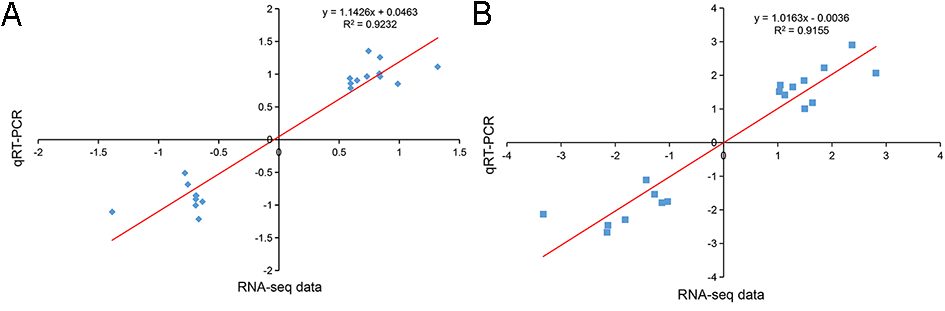


**S6 Fig. Validation of RNA-seq expression data by qRT-PCR analysis**. Validation was performed

using 10 randomly selected DEGs in (A) ND476, and (B) ZX978. The plots demonstrate the

expression ratio in log scale with base of two. The X-axis indicates qRT-PCR log scale; the Y-axis

indicates RNA-seq log scale.
